# Supplementary material for: A conformational switch in clathrin light chain regulates lattice structure and endocytosis at the plasma membrane of mammalian cells
Source: Nat Commun. 2023 Feb 9;14:732. doi: 10.1038/s41467-023-36304-7 (PMC9911608; doi:10.1038/s41467-023-36304-7)
Supplement: Supplementary file 1 — Supplementary Information [file 41467_2023_36304_MOESM1_ESM.pdf]

Supplementary Information

A conformational switch in clathrin light chain regulates lattice structure and endocytosis at the plasma membrane of mammalian cells

Kazuki Obashi, Kem A. Sochacki, Marie-Paule Strub, Justin W. Taraska*

Biochemistry and Biophysics Center, National Heart, Lung, and Blood Institute, National Institutes of Health, 50 South Drive, Building 50, Bethesda, MD 20892, USA.

*Corresponding author. Email: justin.taraska@nih.gov

Supplementary Figure 1: Structural model of clathrin triskelion.

Supplementary Figure 2: FRET pairs used in this study.

Supplementary Figure 3: FLIM-FRET imaging with EGFP and ShadowY at single CCS resolution.

Supplementary Figure 4: Schematic models and simulations for FRET between EGFP-CLC and CLC-ShadowY.

Supplementary Figure 5: Mean fluorescence lifetimes of FRET-CLEM measurements on EGFP and ShadowY attached CLC probes, and FRET-CLEM measurements between N- and N-terminus.

Supplementary Figure 6: Linker flexibility of FRET probes measured with polarized toral internal reflection fluorescence microscopy.

Supplementary Figure 7: Estimation of expression levels in transfected constructs by western blot.

Supplementary Figure 8: Relationship between photon counts and mean fluorescence lifetimes.

Supplementary Figure 9: FRET-CLEM with neuronal CLC.

Supplementary Figure 10: FRET-CLEM with SK-MEL-2 cells.

Supplementary Figure 11: FLIM imaging in living cells.

Supplementary Figure 12: FRET-CLEM with various expression levels in transfected constructs.

Supplementary Figure 13: Mean fluorescence lifetimes of FRET-CLEM measurements with DPA.

Supplementary Figure 14: FRET-CLEM with SNAP-CLC-EGFP and EGFP-CLC-SNAP.

Supplementary Figure 15: FKBP/FRB dimerization was confirmed by changes in probe distributions and FRET efficiencies.

Supplementary Figure 16: PREM Images of cells with manipulation of CLC conformation.

Supplementary Figure 17: Transferrin uptake assay and surface staining of transferrin receptors.

Supplementary Figure 18: FRET simulations at various CLC N-terminal positions in relation to the heavy chain proximal leg domain.

Supplementary Figure 19: PREM analysis on lattice structures.

Supplementary Data 1: Plasmid list used in this study.

Source data are provided in Source Data file.

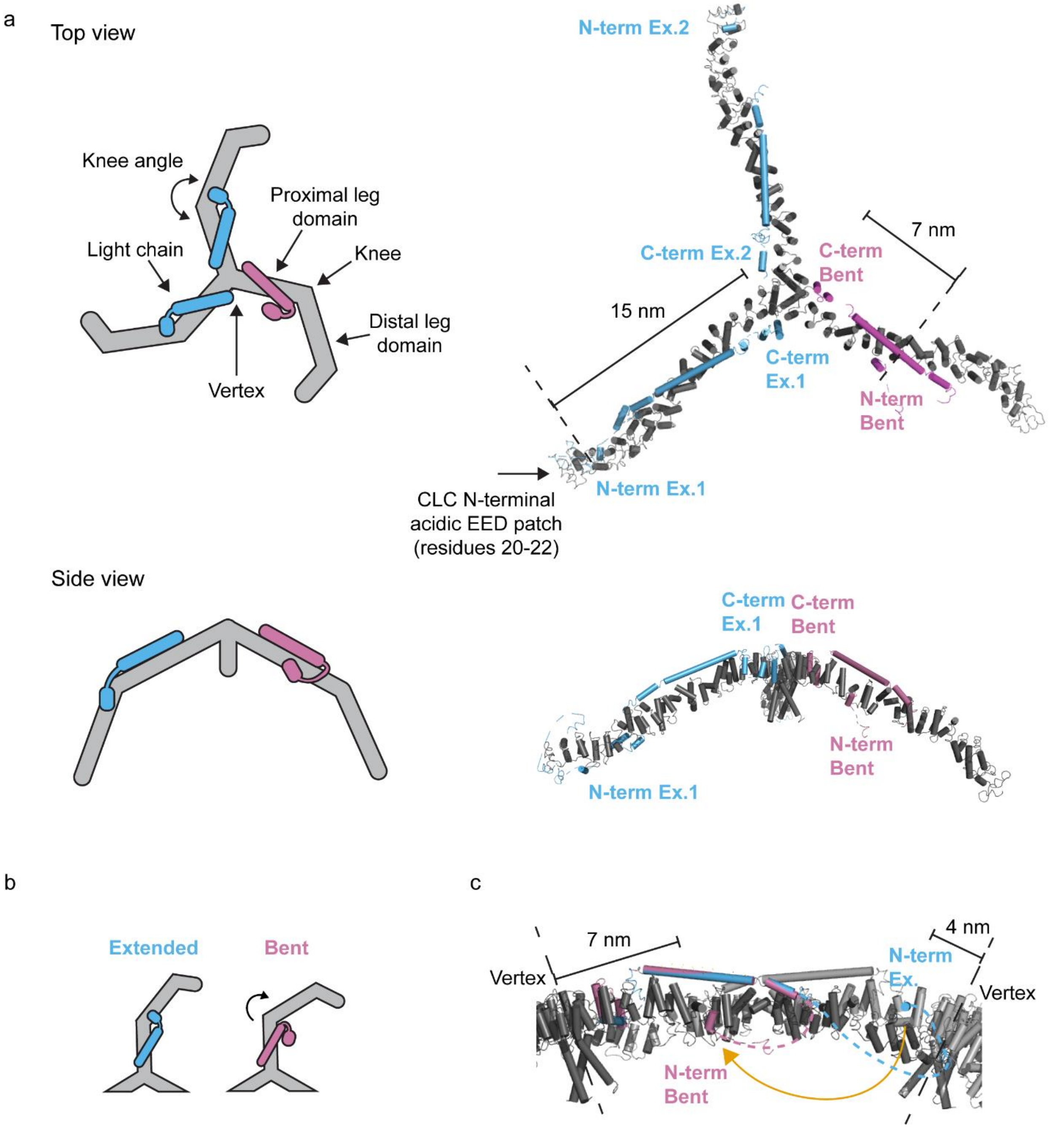

Supplementary Figure 1: Structural model of clathrin triskelion.

(a) A structural model of a clathrin triskelion. Top and side views are shown. The model is based on PDB 3LVG.
(b) Schematic models for conformational changes in CLC and heavy chain knee.
(c) Side view of Fig. 2b. The distances between triskelion vertex (CLC C-terminus) and N-terminus of extended or bent CLC are shown.

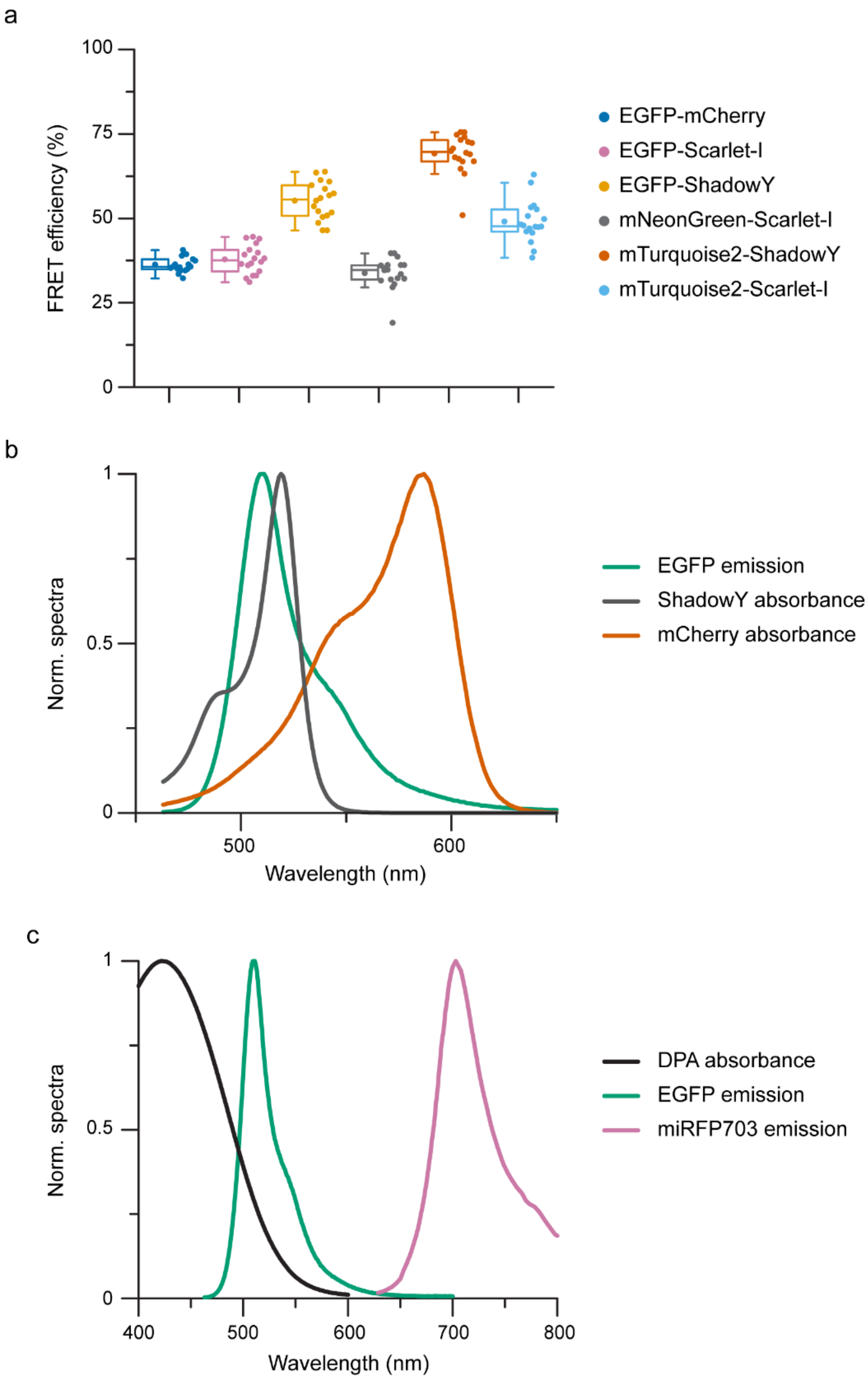

Supplementary Figure 2: FRET pairs used in this study.

(a) FRET efficiencies of tandemly-connected fluorescent proteins (FPs). Fluorescence lifetimes were measured in fixed HeLa cells expressing FPs or tandemly-connected FPs. These probes were expressed in the cytosol. Amplitude-weighted fluorescence lifetimes were estimated from fluorescence lifetime decays by fitting with a bi-exponential. FRET efficiencies were then

calculated from amplitude-weighted fluorescence lifetimes of the donor only and tandem-connected FPs. n = 18 cells from 3 experiments for each condition. For box plots, box is interquartile range, center line is median, center circle is mean, whiskers are minimum and maximum data points with a coefficient value of 1.5.

(b) Normalized spectra of emission of EGFP, absorbance of ShadowY, and absorbance of mCherry.

(c) Normalized spectra of absorbance of DPA, emission of EGFP, and emission of miRFP703. Source data are provided as a Source Data file.

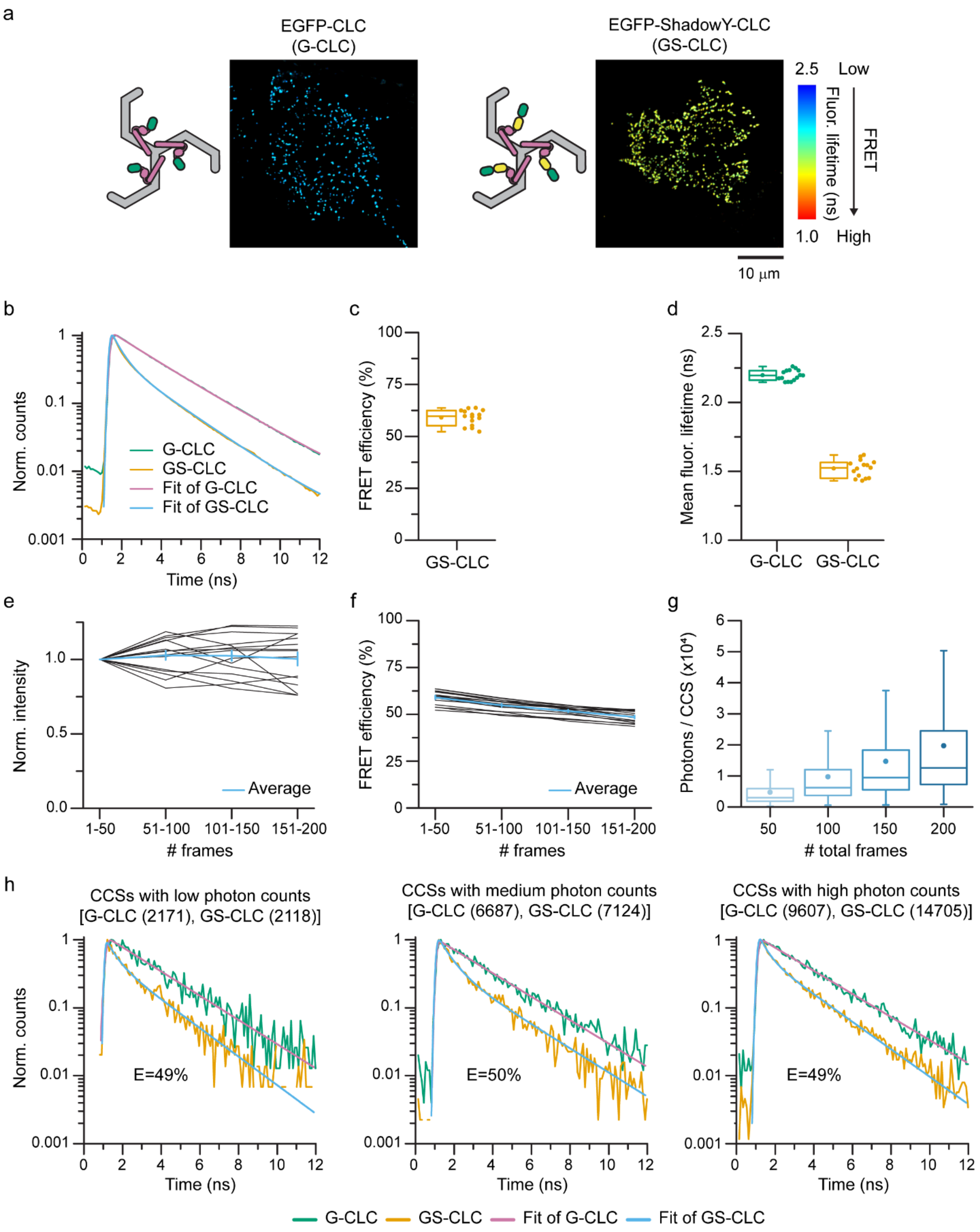

Supplementary Figure 3: FLIM-FRET imaging with EGFP and ShadowY at single CCS resolution.

(a) FLIM images of unroofed membranes of HeLa cells expressing EGFP-CLC (left) or EGFP-ShadowY-CLC (right). n = 3 experiments. Scale 10 μm.
(b) Fluorescence lifetime decays from EGFP-CLC or EGFP-ShadowY-CLC on unroofed membranes. And fluorescence lifetime decays were fitted with a bi-exponential.
(c) FRET efficiencies of EGFP-ShadowY-CLC on unroofed membranes. n = 15 cells from 3 experiments.
(d) Mean fluorescence lifetimes of EGFP-CLC or EGFP-ShadowY-CLC on unroofed membranes. n = 15 cells from 3 experiments for each condition.

(e and f) Unroofed membranes of HeLa cells expressing EGFP-ShadowY-CLC were imaged repeatedly (50 frames, 4 times). Changes in fluorescence intensity (e) and FRET efficiency (f). n = 15 cells from 3 experiments.

(g) Histogram of photon counts per single CCSs on unroofed membrane of HeLa cells expressing EGFP-ShadowY-CLC. n = 2993 CCSs from 15 cells from 3 experiments.
(h) Representable fluorescence lifetime decays of single CCSs on unroofed membranes of HeLa cells expressing EGFP-CLC or EGFP-ShadowY-CLC with different total photon counts indicated in brackets. The fluorescence lifetime decays were fitted with a bi-exponential and FRET efficiencies were estimated.
For box plots, box is interquartile range, center line is median, center circle is mean, whiskers are minimum and maximum data points with a coefficient value of 1.5. Source data are provided as a Source Data file.

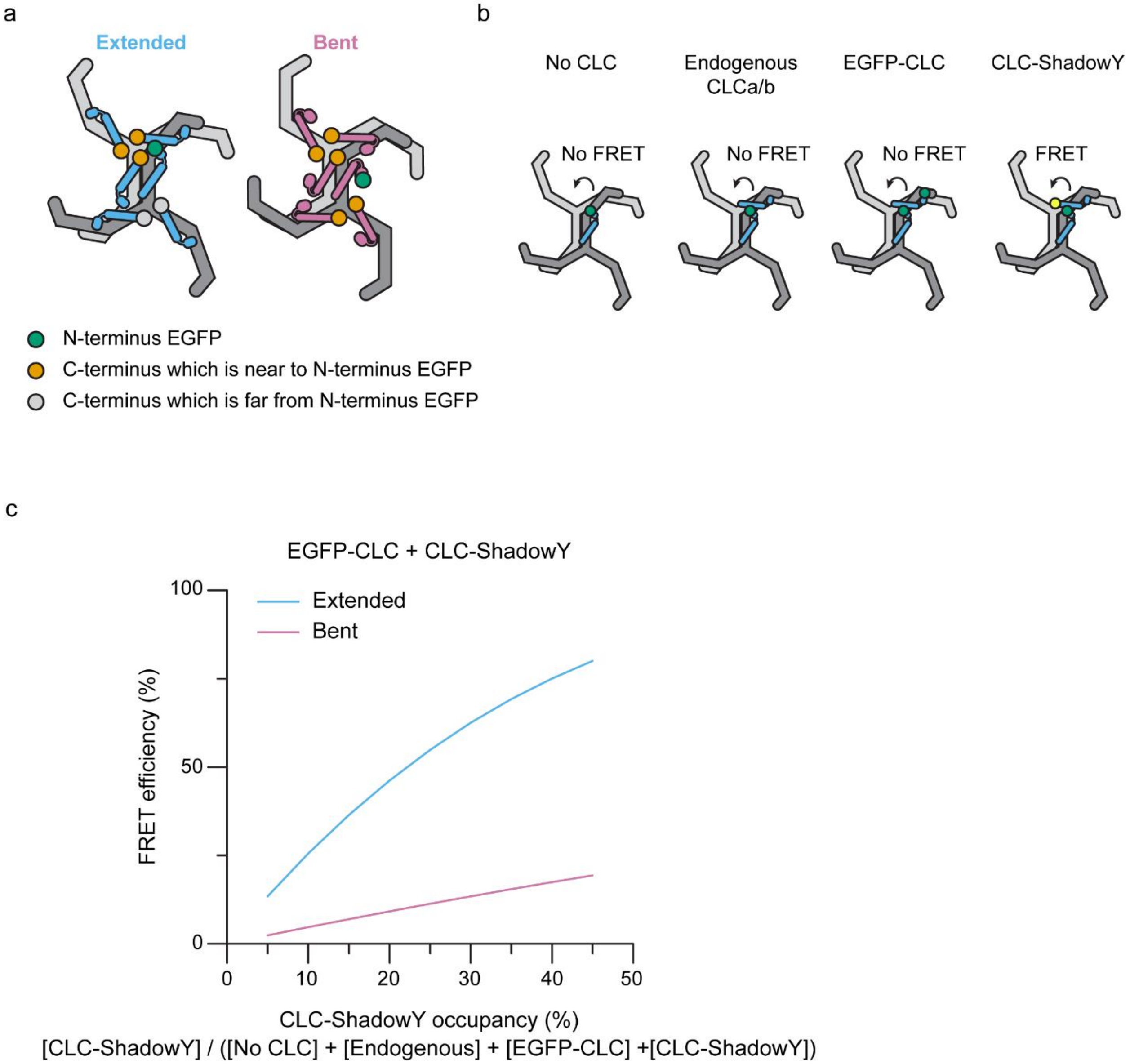

Supplementary Figure 4: Schematic models and simulations for FRET between EGFP-CLC and CLC-ShadowY.

(a) Schematic models of two assembled triskelia with extended (left) or bent CLCs (right). A CLC N-terminus position (green) and C-terminus positions of surrounding CLCs (orange and grey) are shown. In the case of extended conformation, three C-terminal positions (orange positions) are within distances of 45 Å from EGFP of EGFP-CLC and would induce detectable FRET if these positions are occupied by ShadowY. The other two C-terminal positions (grey positions) are outside the range of detectable FRET (greater than 150 Å). On the other hand, in the case of bent conformation, five C-terminal positions (orange positions) locate near EGFP of EGFP-CLC (65-120 Å) and would induce detectable FRET if these positions are occupied by ShadowY.

(b) Four CLC-binding/unbinding states in clathrin heavy chain. If we focus on single clathrin heavy chain, there are four different states; without CLC, endogenous CLCa or CLCb binding, EGFP-CLC binding, or CLC-ShadowY binding.

(c) Simulation of FRET between EGFP-CLC and CLC-ShadowY at various expression levels. We only considered the FRET among EGFP-CLC and the closest five C-terminal positions because other C-terminal positions would be outside the range of detectable FRET. In addition, we assumed that FP-attached CLCs are randomly incorporated throughout the clathrin lattices. We previously revealed that density of FP-attached CLC in CCSs does not differ among clathrin structures with different curvatures[4]. So it is reasonable that the degree of FP-tagged CLC binding to clathrin heavy chains in clathrin lattices is not different across all clathrin subtypes. We defined the probability of clathrin heavy chain binding to CLC-ShadowY as CLC-ShadowY occupancy rate. This number should mirror the expression levels of CLC-ShadowY and the FRET efficiency increases as CLC-ShadowY occupancy increases. FRET efficiency of extended conformation of the light chain is always larger than the bent conformation at all possible CLC-ShadowY occupancies. Thus, conformational changes in CLC can be measured among CCSs across a range of expression levels (occupancies and stoichiometries) because all possible states show a decrease in FRET from the extended state to the bent state.

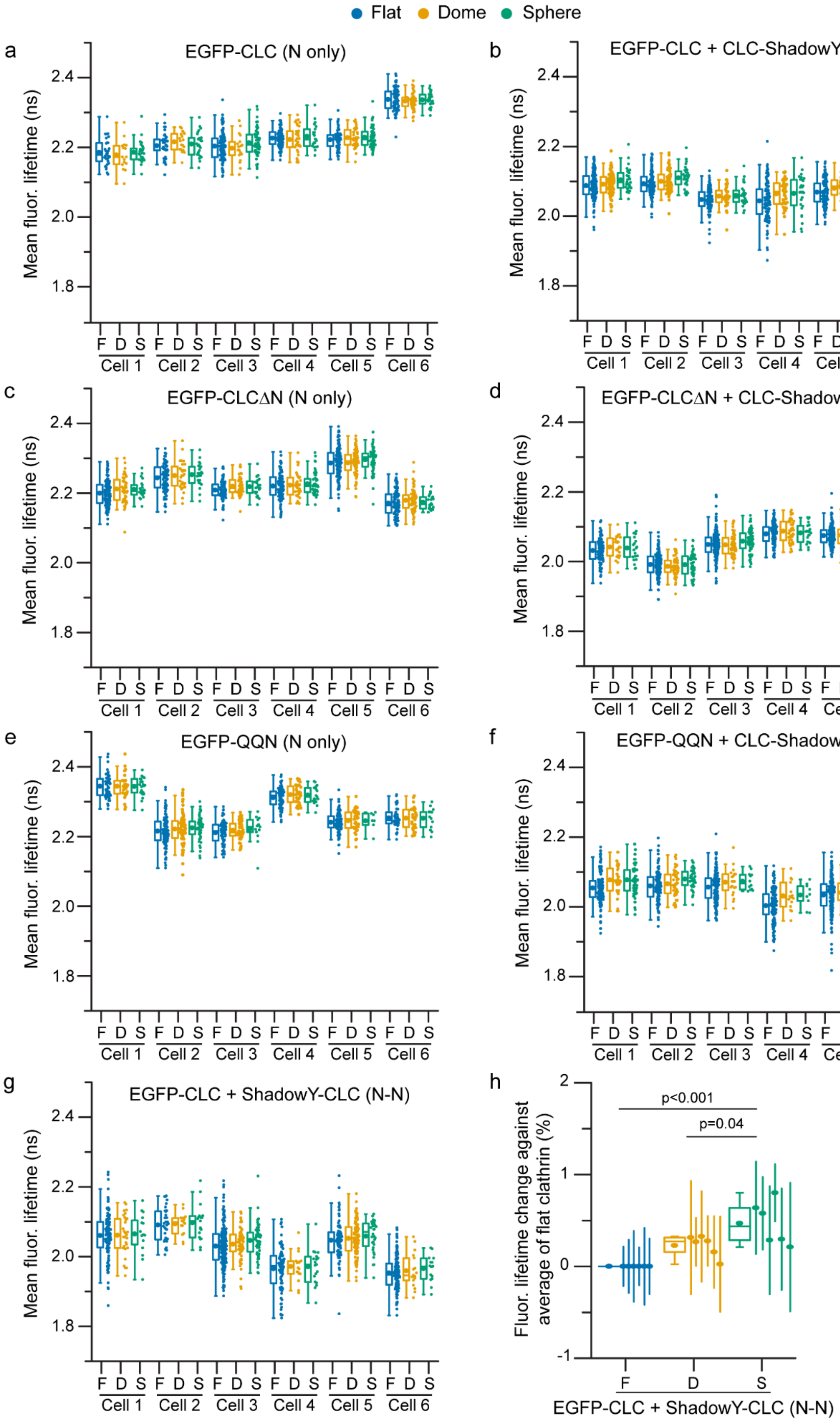

Flat
Dome
Sphere
a
EGFP-CLC (N only)
Mean fluor. lifetime (ns)
2.4
2.2
2.0
1.8
F D S F D S F D S F D S F D S F D S
Cell 1 Cell 2 Cell 3 Cell 4 Cell 5 Cell 6
b
EGFP-CLC + CLC-ShadowY (N-C)
c
EGFP-CLCΔN (N only)
d
EGFP-CLCΔN + CLC-ShadowY (N-C)
e
EGFP-QQN (N only)
f
EGFP-QQN + CLC-ShadowY (N-C)
g
EGFP-CLC + ShadowY-CLC (N-N)
h
Fluor. lifetime change against average of flat clathrin (%)
2
1
0
-1
p<0.001
p=0.04
F D S
EGFP-CLC + ShadowY-CLC (N-N)

Supplementary Figure 5: Mean fluorescence lifetimes of FRET-CLEM measurements on EGFP and ShadowY attached CLC probes, and FRET-CLEM measurements between N- and N-terminus.

(a-f) Mean fluorescence lifetimes from single CCSs of experiments in Fig. 2. *n* (flat, domed, sphere CCSs/cell) = (30-107, 16-49, 22-53) for EGFP-CLC (a), (82-163, 32-77, 22-32) for EGFP-CLC and CLC-ShadowY (b), (78-128, 28-76, 22-55) for EGFP-CLCΔN (c), (94-166, 18-74, 13-60) for EGFP-CLCΔN and CLC-ShadowY (d), (42-129, 26-69, 7-49) for EGFP-QQN (e), and (107-183, 15-52, 9-68) for EGFP-QQN and CLC-ShadowY (f). Number of experiments and samples were described in the legend of Fig. 2.

(g, h) FRET-CLEM was performed on HeLa cells expressing EGFP-CLC and ShadowY-CLC. Mean fluorescence lifetimes from single CCSs (g) and their changes (h) were analyzed by categorizing them according to lattice structures (flat, domed and sphere). For plot H, each dot is from one cell experiment and errors are SE. *n* = 6 cells from 3 experiments, *n* (flat, domed, sphere CCSs/cell) = (32-202, 15-88, 15-56). One-way ANOVA, then Tukey's test.

For box plots, box is interquartile range, center line is median, center circle is mean, whiskers are minimum and maximum data points with a coefficient value of 1.5. Source data are provided as a Source Data file.

a

		Sequence<br>Nup54 Linker EGFP
Rigid linker	Nup54-EGFP494(0)	DIKLVEAAAKEELFT
	Nup54-EGFP494(1)	DIKLVAEAAAKEELFT
Flexible linker	Nup54-EGFP494(flex0)	DIKLVGGGGSEELFT
	Nup54-EGFP494(flex1)	DIKLVGGGGSSEELFT

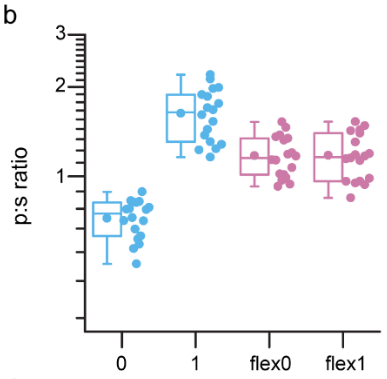

c

		Sequence<br>CLC Linker EGFP
Rigid linker	CLC-EGFP206(5)	LMSKQEAAAKEELFT
	CLC-EGFP206(6)	LMSKQAEAAAKEELFT
	CLC-EGFP206(7)	LMSKQAEAAAKEEELFT
Flexible linker	CLC-EGFP206(flex5)	LMSKQGGGGSEELFT
	CLC-EGFP206(flex6)	LMSKQGGGGSSEELFT
	CLC-EGFP206(flex7)	LMSKQGGGGSSGEELFT

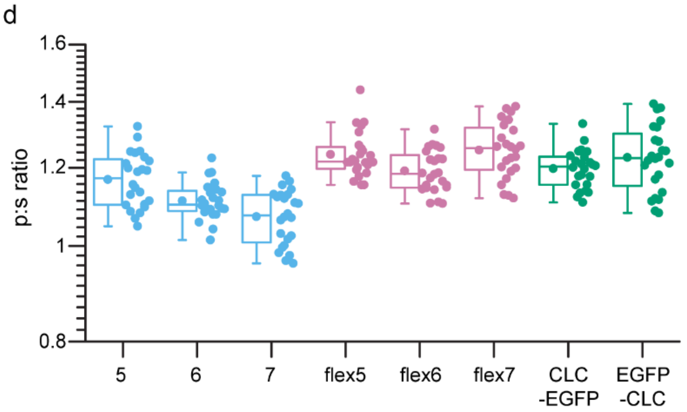

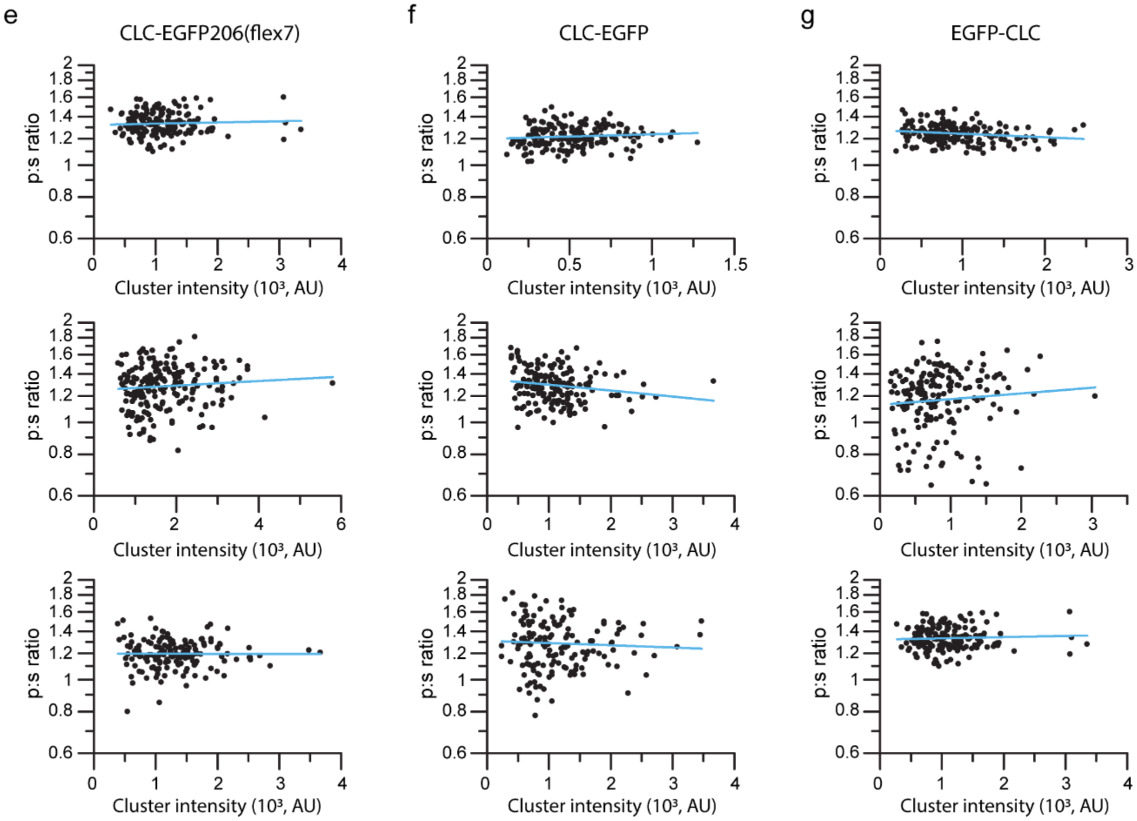

Supplementary Figure 6: Linker flexibility of FRET probes measured with polarized toral internal reflection fluorescence microscopy.

(a) Nucleoporin (Nup54) constructs with rigid or flexible linkers between Nup54 and EGFP. The alpha helix at the N terminus of EGFP (from residue 5) was conjugated to the C terminus of Nup54 domains at residue 494 with different linkers.
(b) The p:s ratio of Nup54-EGFP494 constructs. Each dot is from one cell. The value changed for rigid linkers by changing the linker length. However, this did not happen for the constructs with flexible linkers. *n* = 18 cells from 3 experiments for each conditions.
(c) CLC constructs with rigid or flexible linkers between CLC and EGFP. The alpha helix at the N terminus of EGFP (from residue 5) was conjugated to the C terminus of CLC domains at residue 206 with different linkers.
(d) The p:s ratio of CLC-EGFP206 constructs, CLC-EGFP, and EGFP-CLC. Each dot is from one cell. The value changed for rigid linkers by changing the linker length. However, this did not happen for the constructs with flexible linkers. And the p:s values were similar among probes with flexible linkers and FRET constructs (CLC-EGFP and EGFP-CLC). *n* = 24 cells from 3 experiments for each conditions.

(e-g) Examples of the relationship between cluster intensity (arbitrary units) and p:s ratio in single cluster from 3 cells from 3 independent experiments for CLC-EGFP206(flex7) (e), ELC-EGFP (f), and EGFP-CLC (g). *n* = 127-196 clusters for each conditions.

For box plots, box is interquartile range, center line is median, center circle is mean, whiskers are minimum and maximum data points with a coefficient value of 1.5. Source data are provided as a Source Data file.

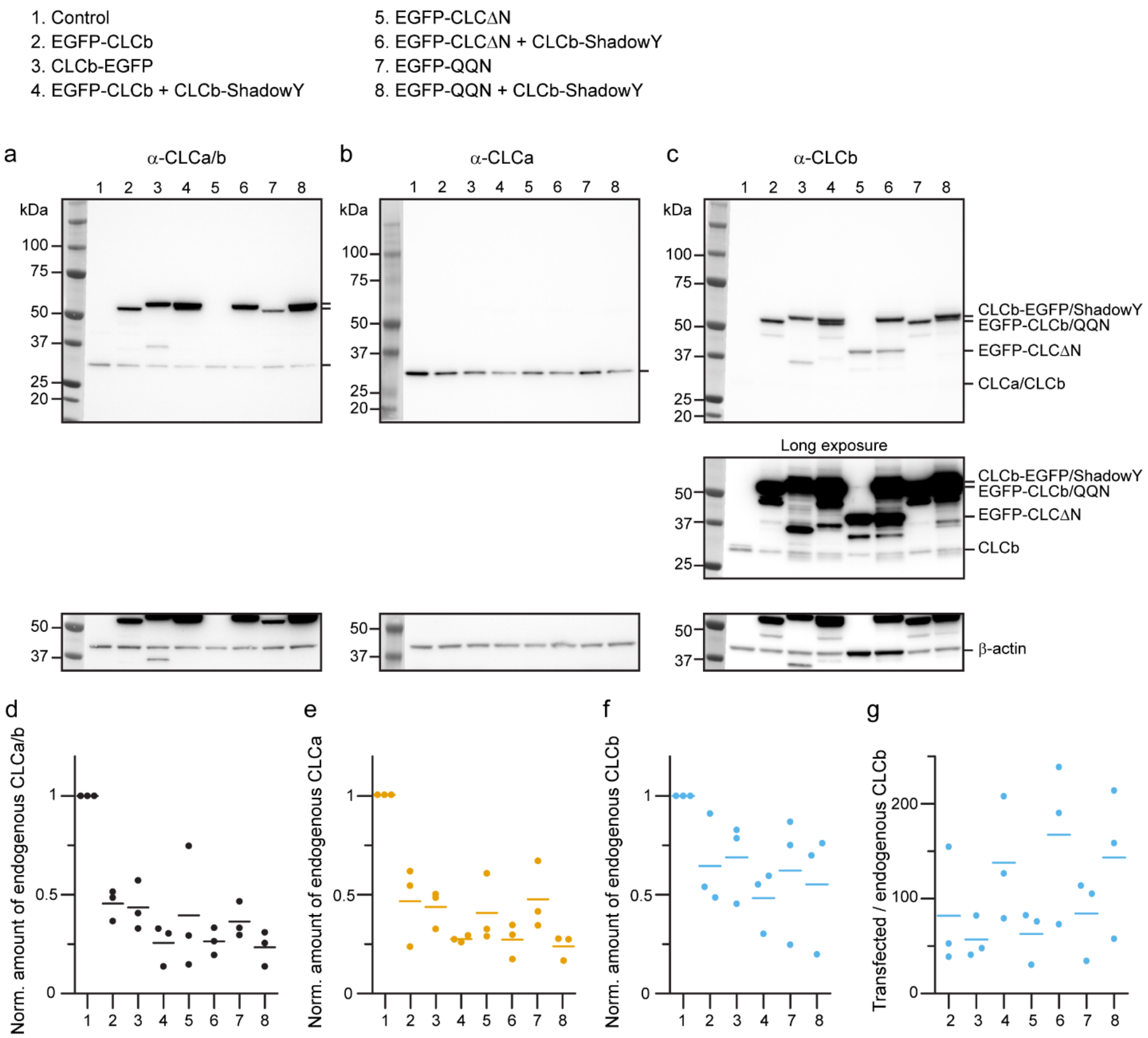

h

	CLCa	CLCb	Transfected CLC	Total CLC	Ratio (Transfected/endogenous)
Control	0.8	0.2	0	1	
Single transfection	0.2	0.1	8	8.3	26.7
Double transfection	0.04	0.06	10.8	10.9	108

Supplementary Figure 7: Estimation of expression levels in transfected constructs by western blot.

(a) Immunoblot with CLCa/b antibody of non-transfected and transfected HeLa cells.

(b) Immunoblot with CLCa antibody of non-transfected and transfected HeLa cells.

(c) Immunoblot with CLCb antibody of non-transfected and transfected HeLa cells.

(d-f) Normalized amount of endogenous CLCa/b (d), CLCa (e), and CLCb (f) in non-transfected and transfected HeLa cells. *n* = 3 experiments for each conditions.

(g) Amount ratio between transfected and endogenous CLCb.

(h) Estimation of expression levels in transfected constructs based on immunoblots results under assumptions that an endogenous amount ratio between CLCa and CLCb is 4:1 and transfection efficiency is 80%.
Source data are provided as a Source Data file.

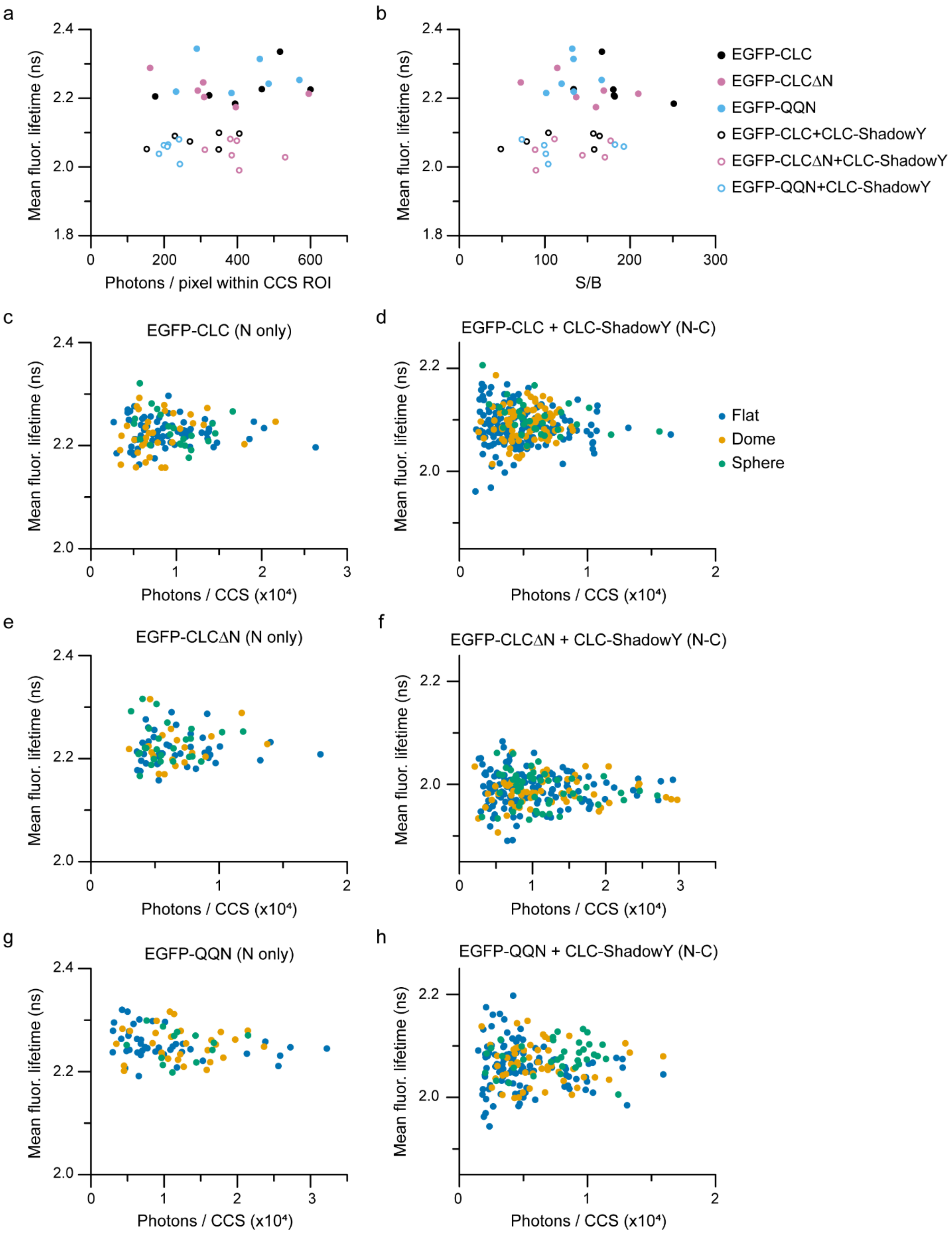

Supplementary Figure 8: Relationship between photon counts and mean fluorescence lifetimes.

(a,b) Relationship between photon counts per pixel (a) or signal-to-background ratio (b) and mean fluorescence lifetimes in clathrin-coated structure ROIs (CCS ROIs). Each dot is from one cell experiment. FRET-CLEM data are from Fig. 2d-f.

(c-h) Representable relationships between photon counts and mean fluorescence lifetimes in single clathrin-coated structures. Single cell data of each condition are from Fig. 2d-f.

Source data are provided as a Source Data file.

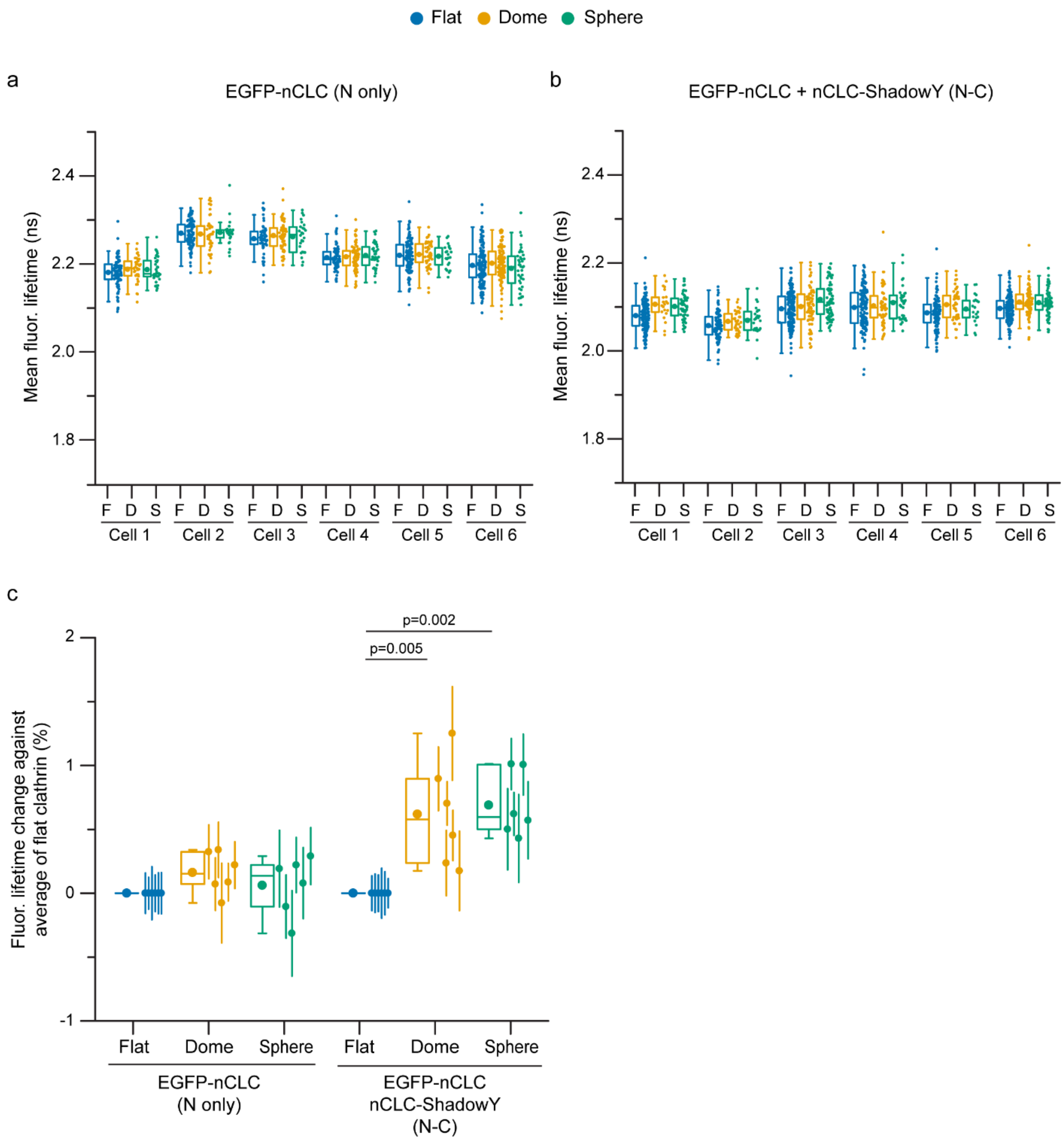

Supplementary Figure 9: FRET-CLEM with neuronal CLC.

FRET-CLEM was performed on HeLa cells expressing either EGFP-nCLC (neuronal isoform), or EGFP-nCLC and nCLC-ShadowY. Mean fluorescence lifetimes from single CCSs (a, b) and their changes (c) were analyzed by categorizing them according to lattice structures (flat, domed and sphere). For plot c, each dot is from one cell experiment and errors are SE. *n* = 6 cells from 3 experiments for each condition, *n* (flat, domed, sphere CCSs/cell) = (56-152, 38-99, 22-39) for EGFP-nCLC (a) and (105-181, 21-81, 20-71) for EGFP-nCLC and nCLC-ShadowY (b). One-way ANOVA, then Tukey's test. For box plots, box is interquartile range, center line is median, center circle is mean, whiskers are minimum and maximum data points with a coefficient value of 1.5. Source data are provided as a Source Data file.

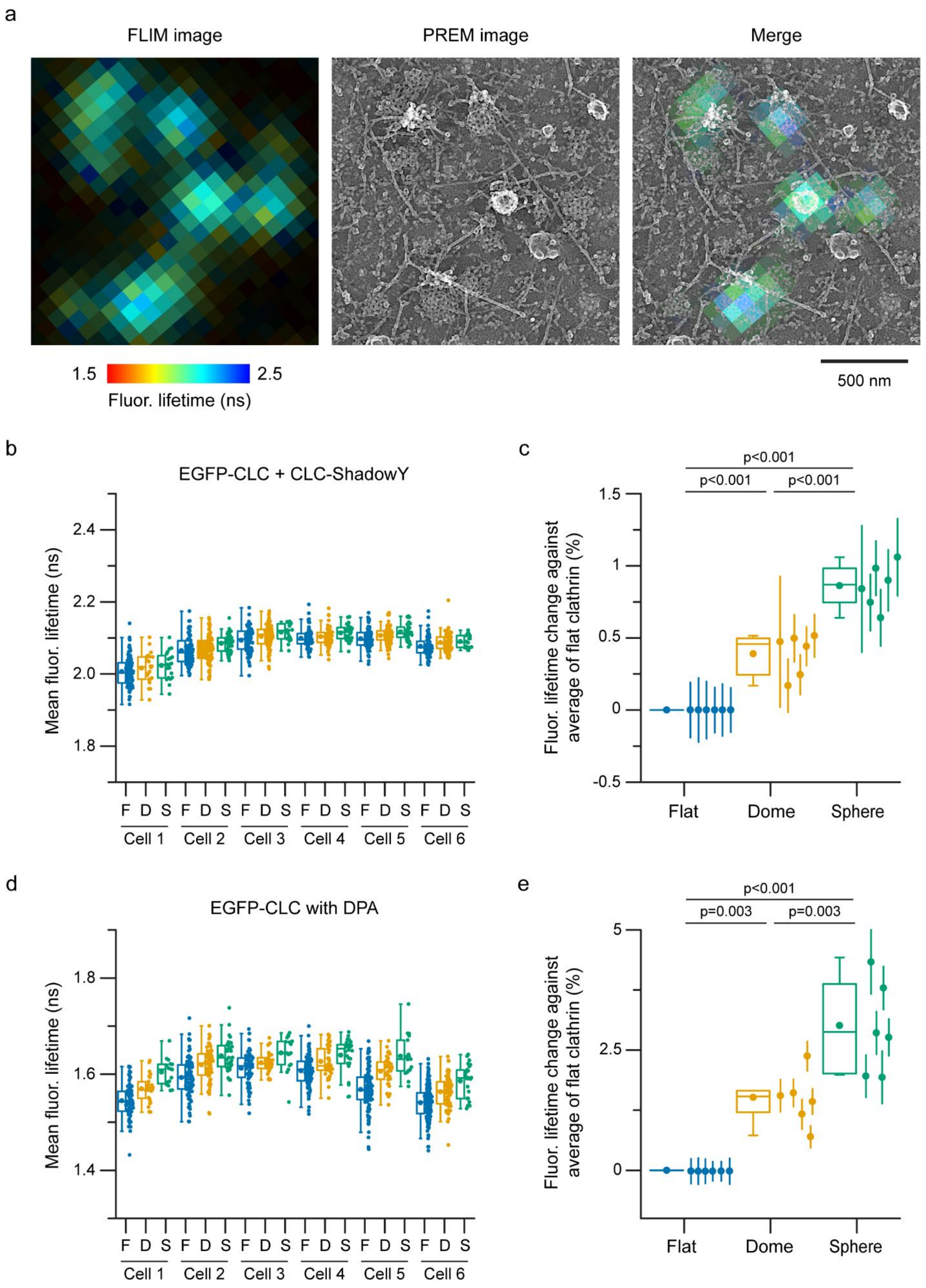
a
FLIM image
PREM image
Merge
1.5
2.5
Fluor. lifetime (ns)
500 nm
b
EGFP-CLC + CLC-ShadowY
Mean fluor. lifetime (ns)
F D S F D S F D S F D S F D S F D S
Cell 1 Cell 2 Cell 3 Cell 4 Cell 5 Cell 6
c
p<0.001
p<0.001
p<0.001
Fluor. lifetime change against average of flat clathrin (%)
Flat
Dome
Sphere
d
EGFP-CLC with DPA
Mean fluor. lifetime (ns)
Cell 1 Cell 2 Cell 3 Cell 4 Cell 5 Cell 6
e
p<0.001
p=0.003
p=0.003
Fluor. lifetime change against average of flat clathrin (%)
Flat
Dome
Sphere

Supplementary Figure 10: FRET-CLEM with SK-MEL-2 cells.

(a) Correlative FLIM-FRET and PREM images of an unroofed membrane of SK-MEL-2 cell expressing EGFP-CLC and CLC-ShadowY. FLIM image (left; photon counts are represented by brightness and fluorescence lifetimes are represented by pseudo color), PREM image (center), and merge image (right). *n* = 5 experiments. Scale 500 nm.

(b,c) FRET-CLEM was performed on SK-MEL-2 cells expressing EGFP-CLC and CLC-ShadowY. Mean fluorescence lifetimes from single CCSs (b) and their changes (c) were analyzed by categorizing them according to lattice structures (flat, domed and sphere). For plot c, each dot is from one cell and errors are SE. *n* = 6 cells from 5 experiments, *n* (flat, domed, sphere CCSs/cell) = (57-107, 22-93, 18-41). One-way ANOVA, then Tukey's test.

(d, e) FRET-CLEM was performed on SK-MEL-2 cells expressing EGFP-CLC with 80 μM of DPA. Mean fluorescence lifetimes from single CCSs (d) and their changes (e) were analyzed by categorizing them according to lattice structures (flat, domed and sphere). For plot e, each dot is from one cell and errors are SE. *n* = 6 cells from 3 experiments, *n* (flat, domed, sphere CCSs/cell) = (66-128, 27-63, 16-33). One-way ANOVA, then Tukey's test.

For box plots, box is interquartile range, center line is median, center circle is mean, whiskers are minimum and maximum data points with a coefficient value of 1.5. Source data are provided as a Source Data file.

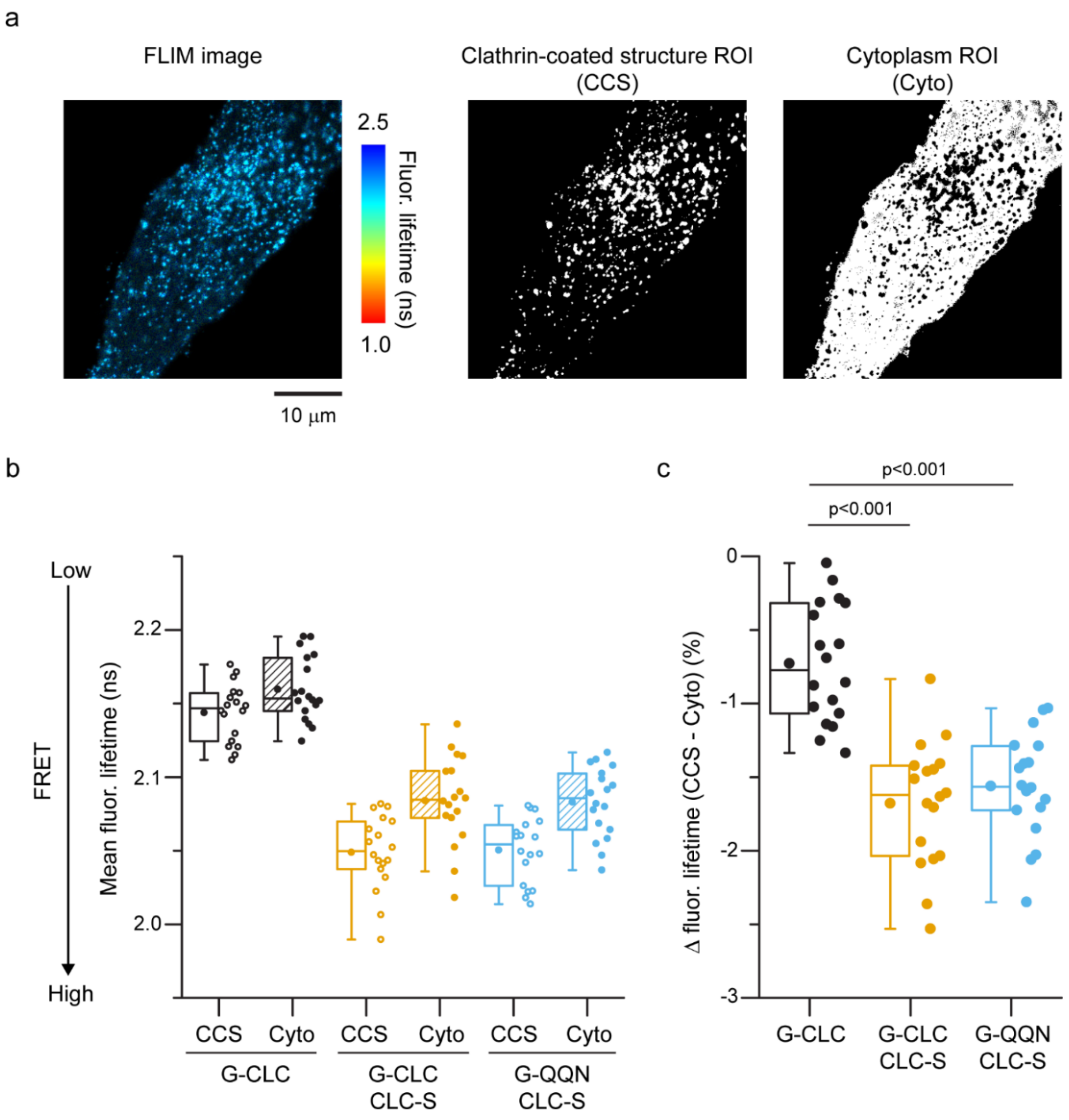

Supplementary Figure 11: FLIM imaging in living cells.

(a) A FLIM image of a living HeLa cell expressing EGFP-CLC (left; photon counts are represented by brightness and fluorescence lifetimes are represented by pseudo color). And ROIs for CCSs (CCS, center) and cytoplasm (Cyto, right) are shown. Scale 10 μm.

(b) Mean fluorescence lifetimes within CCS ROI or Cyto ROI of EGFP-CLC (G-CLC), EGFP-CLC and CLC-ShadowY (G-CLC + CLC-S), or EGFP-QQN and CLC-ShadowY (G-QQN + CLC-S) expressing cells. *n* = 18 cells from 3 experiments for each conditions.

(c) Differences in mean fluorescence lifetimes (shown in panel b) between CCS ROI and Cyto ROI. One-way ANOVA, then Tukey's test.
For box plots, box is interquartile range, center line is median, center circle is mean, whiskers are minimum and maximum data points with a coefficient value of 1.5. Source data are provided as a Source Data file.

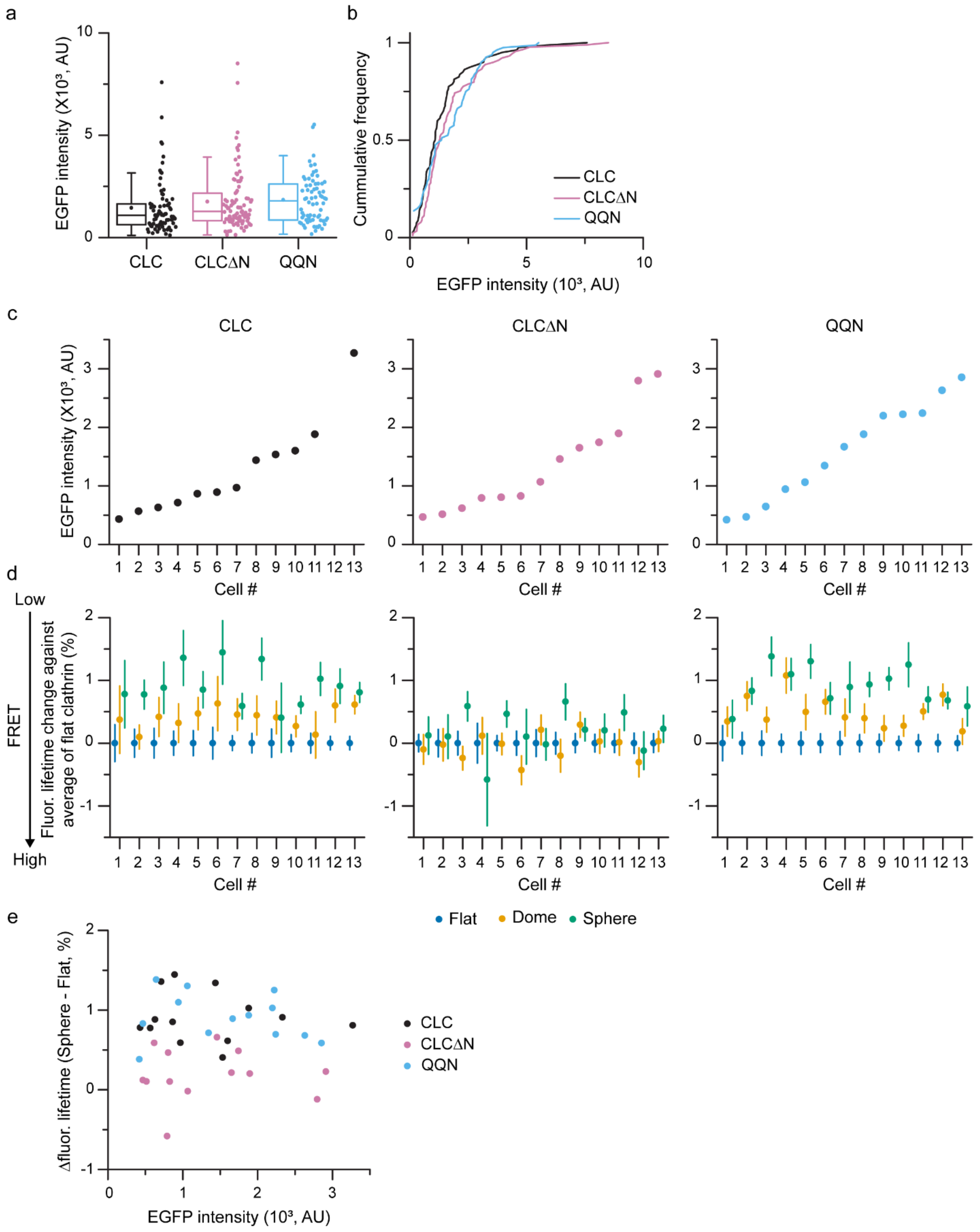

Supplementary Figure 12: FRET-CLEM with various expression levels in transfected constructs.

(a,b) Cell average EGFP intensities (arbitrary units) (a) and their cummulative friquencies (b) in unroofed HeLa cells expressiong EGFP-CLC and CLC-ShadowY (CLC), EGFP-CLCΔN and CLC-ShadowY (CLCΔN), or EGFP-QQN and CLC-ShadowY (QQN). *n* = 78 (CLC), 88 (CLCΔN), and 69 cells (QQN) from 7 experiments for each conditions. For box plots, box is interquartile range, center line is median, center circle is mean, whiskers are minimum and maximum data points with a coefficient value of 1.5.

(c) Cell average EGFP intensities in unroofed HeLa cells used in FRET-CLEM analysis. *n* = 13 cells from 5 (CLC), 6 (CLCΔN), and 5 experiments (QQN).
(d) Mean fluorescence lifetimes from single CCSs were analyzed by categorizing them according to lattice structures (flat, domed and sphere) and they were compared to the average values of flat structures. Errors are SE.
(e) Relationship between cell average EGFP intensities and degree of fluorescence lifetime changes between sphere and flat structures.
Source data are provided as a Source Data file.

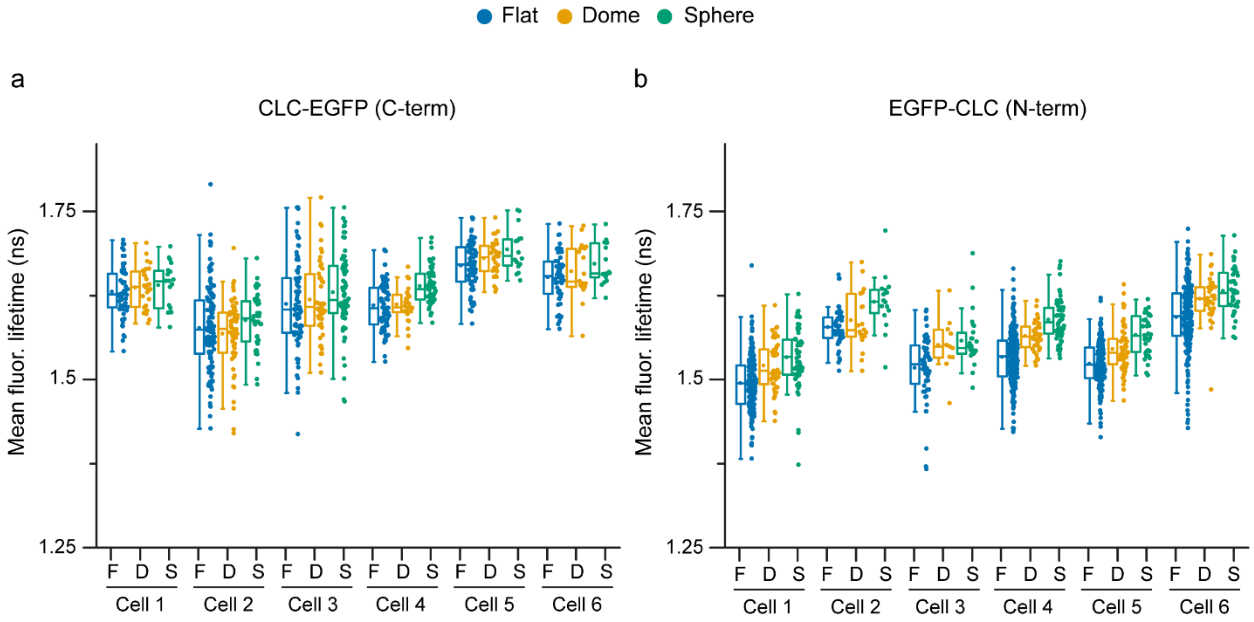

Supplementary Figure 13: Mean fluorescence lifetimes of FRET-CLEM measurements with DPA.

Mean fluorescence lifetimes from single CCSs of experiments in Fig. 3e. *n* (flat, domed, sphere CCSs/cell) = (45-239, 11-59, 16-65) for CLC-EGFP (a) and (52-128, 19-58, 16-72) for EGFP-CLC (b). For box plots, box is interquartile range, center line is median, center circle is mean, whiskers are minimum and maximum data points with a coefficient value of 1.5. Number of experiments and samples were described in the legend of Fig. 3. Source data are provided as a Source Data file.

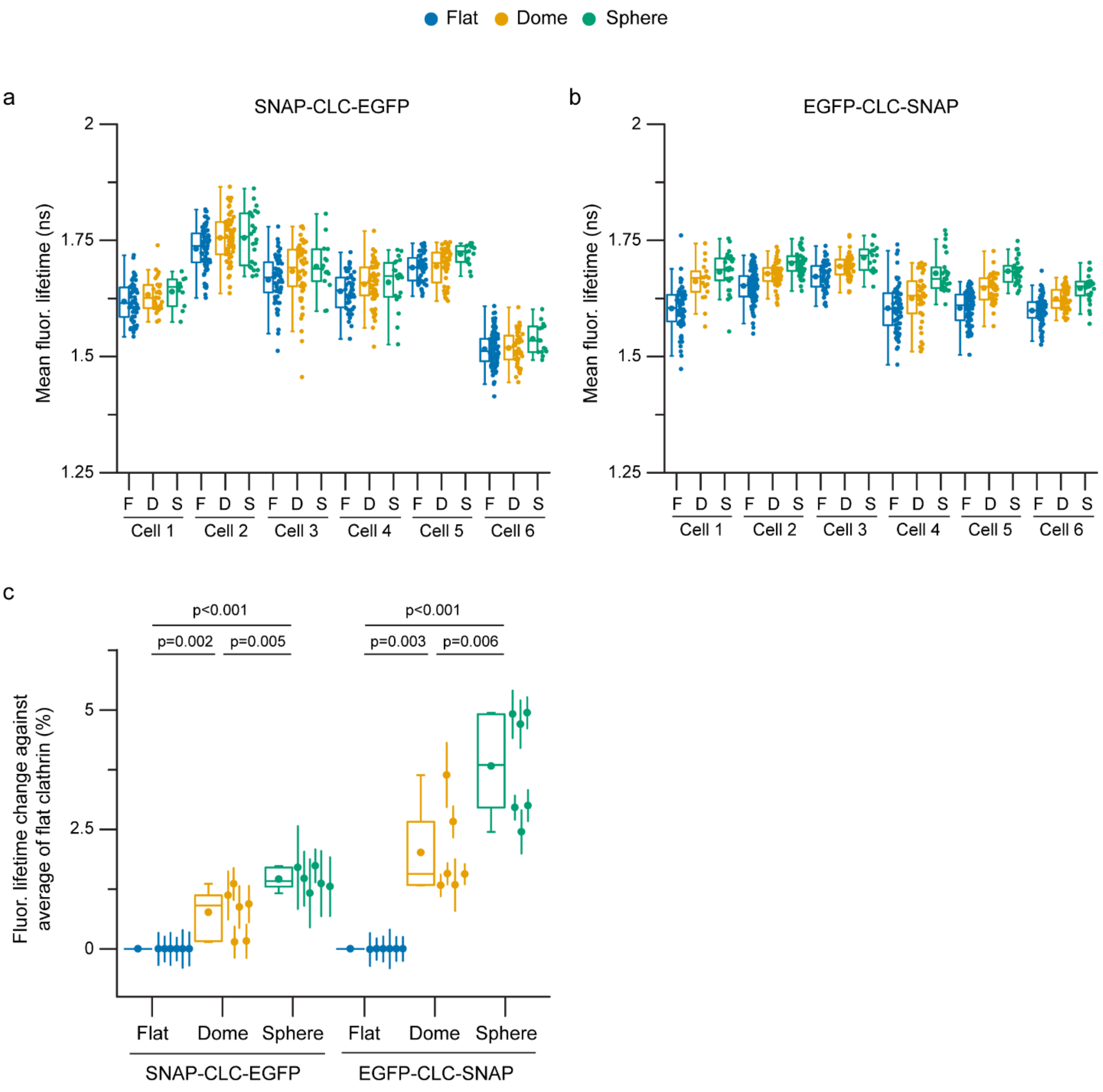

Supplementary Figure 14: FRET-CLEM with SNAP-CLC-EGFP and EGFP-CLC-SNAP.

FRET-CLEM was performed on HeLa cells expressing either SNAP-CLC-EGFP or EGFP-CLC-SNAP with DPA. DPA concentrations were 3 μM for SNAP-CLC-EGFP and 80 μM for EGFP-CLC-SNAP to obtain ~50% FRET efficiencies to make the degree of fluorescence lifetime changes similar. Mean fluorescence lifetimes from single CCSs (a, b) and their changes (c) were analyzed by categorizing them according to lattice structures (flat, domed and sphere). For plot c, each dot is from one cell and errors are SE. *n* = 6 cells from 3 experiments for each condition, *n* (flat, domed, sphere CCSs/cell) = (48-101, 28-71, 11-24) for SNAP-CLC-EGFP (a) and (49-95, 17-51, 16-40) for EGFP-CLC-SNAP (b). One-way ANOVA, then Tukey's test. For box plots, box is interquartile range, center line is median, center circle is mean, whiskers are minimum and maximum data points with a coefficient value of 1.5. Source data are provided as a Source Data file.

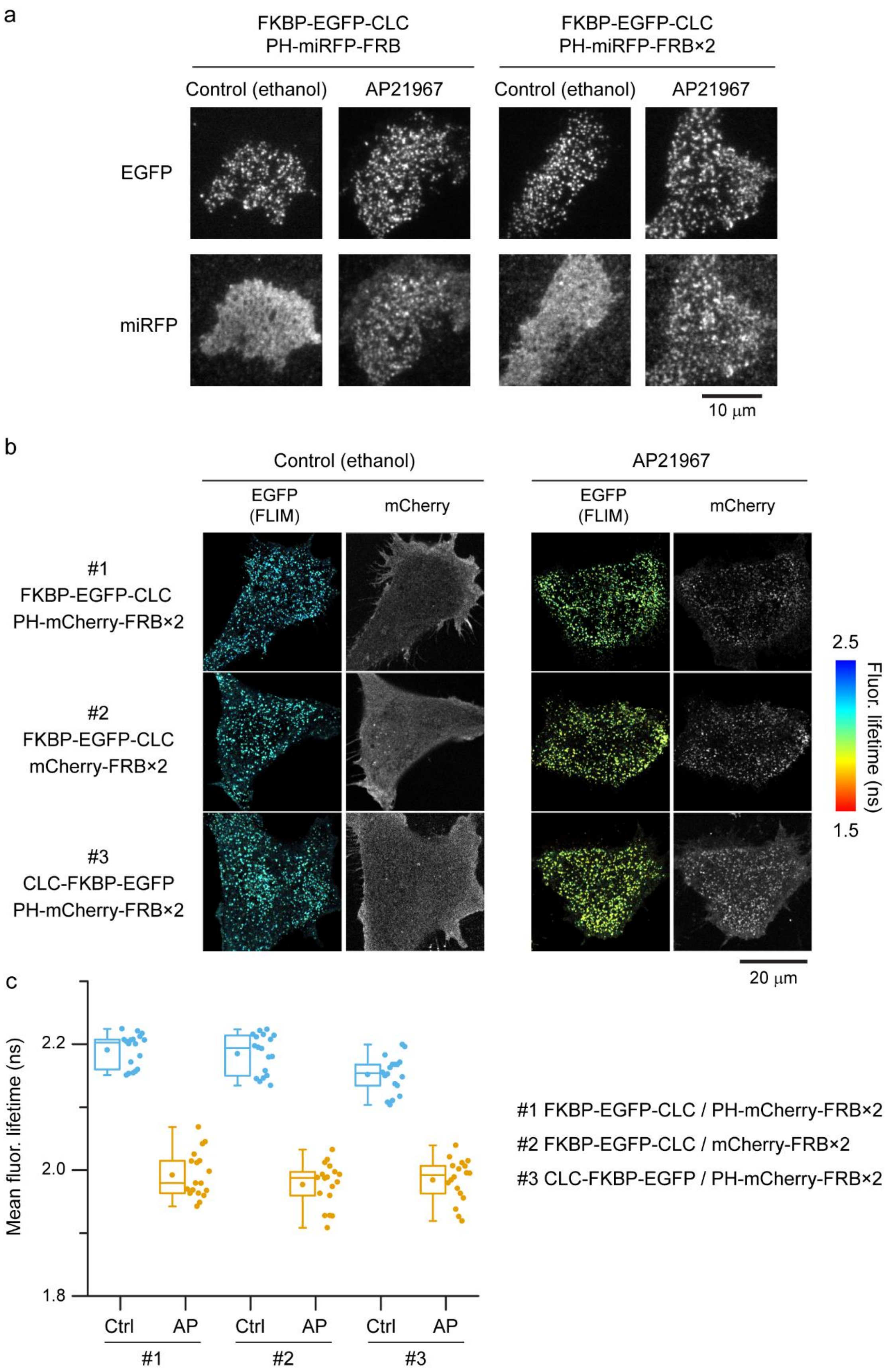
a
FKBP-EGFP-CLC
PH-miRFP-FRB
FKBP-EGFP-CLC
PH-miRFP-FRB×2
Control (ethanol)
AP21967
Control (ethanol)
AP21967
EGFP
miRFP
10 μm
b
Control (ethanol)
AP21967
EGFP
(FLIM)
mCherry
EGFP
(FLIM)
mCherry
#1
FKBP-EGFP-CLC
PH-mCherry-FRB×2
#2
FKBP-EGFP-CLC
mCherry-FRB×2
#3
CLC-FKBP-EGFP
PH-mCherry-FRB×2
2.5
Fluor. lifetime (ns)
1.5
20 μm
c
Mean fluor. lifetime (ns)
2.2
2.0
1.8
Ctrl
AP
Ctrl
AP
Ctrl
AP
#1
#2
#3
#1 FKBP-EGFP-CLC / PH-mCherry-FRB×2
#2 FKBP-EGFP-CLC / mCherry-FRB×2
#3 CLC-FKBP-EGFP / PH-mCherry-FRB×2

Supplementary Figure 15: FKBP/FRB dimerization was confirmed by changes in probe distributions and FRET efficiencies.

(a) TIRF images of unroofed membranes of HeLa cells expressing FKBP-EGFP-CLC and either PH-miRFP-FRB (left) or PH-miRFP-FRB×2 (right). Cells were unroofed after 15 min incubation with AP21967 or ethanol (control). Scale 10 μm.

(b) FLIM and confocal images of fixed HeLa cells expressing FKBP and FRB probes treated with AP21967 or ethanol (control). Scale 20 μm.

(c) Mean fluorescence lifetimes of fixed HeLa cells expressing FKBP and FRB probes treated with AP21967 or ethanol (control). n = 18 cells from 3 experiments for each conditions. For box plots, box is interquartile range, center line is median, center circle is mean, whiskers are minimum and maximum data points with a coefficient value of 1.5.
Source data are provided as a Source Data file.

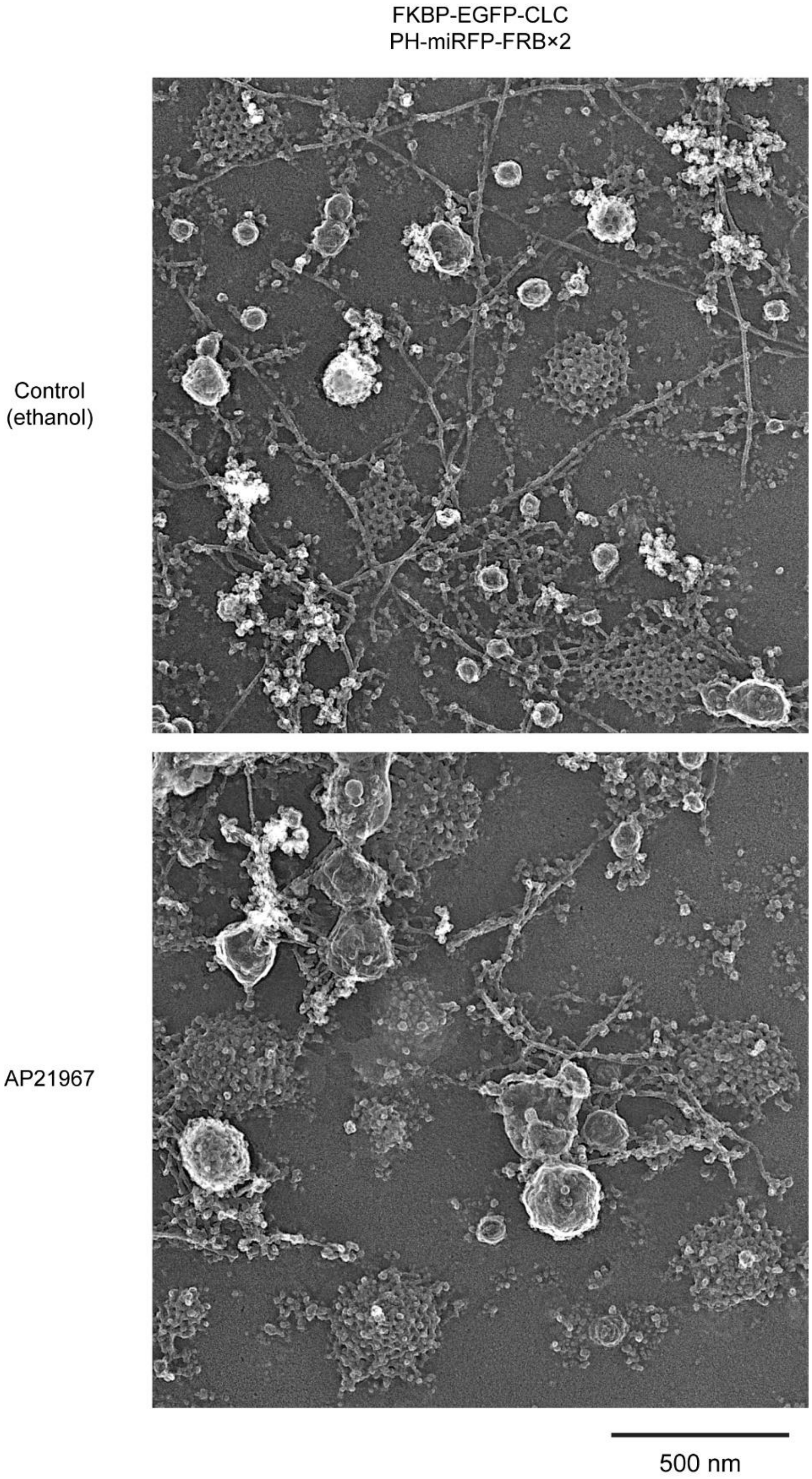

Supplementary Figure 16: PREM Images of cells with manipulation of CLC conformation.

PREM images of unroofed membranes of HeLa cells expressing FKBP-EGFP-CLC with PH-miRFP-FRB×2. Cells were treated with ethanol (control, top) or AP21967 (bottom) for 15 minutes before unroofing. *n* = 3 experiments for each conditions. Scale 500 nm.

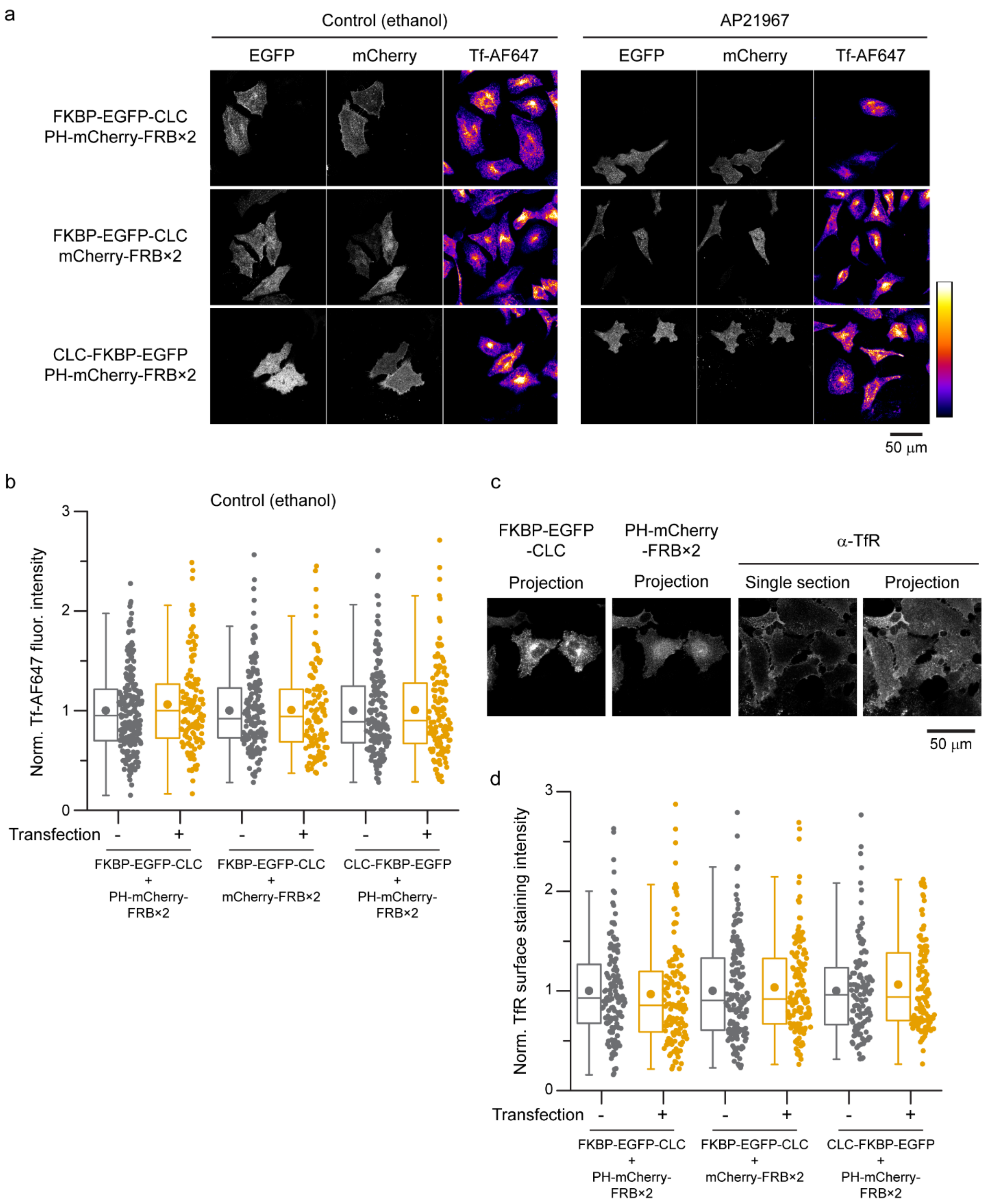

Supplementary Figure 17: Transferrin uptake assay and surface staining of transferrin receptors.

(a) Confocal projection images of EGFP, mCherry, and Alexa Fluor 647 conjugated transferrin (Tf-AF647) in HeLa cells expressing FKBP and FRB probes treated with AP21967 or ethanol (control). Fluorescence intensity of Tf-AF647 (arbitrary units) is represented by pseudo color. Scale 50 μm.

(b) Transferrin uptake in HeLa cells expressing FKBP and FRB probes treated with ethanol (control). Fluorescence intensities of incorporated Tf-AF647 normalized by non-transfected cells in the same sample were compared between non-transfected and transfected cells. *n* = 202 (non-transfected, FKBP-EGFP-CLC+PH-mCherry-FRB2), 119 (transfected, FKBP-EGFP-CLC+PH-mCherry-FRB2), 154 (non-transfected, FKBP-EGFP-CLC+mCherry-FRB2), 106 (transfected, FKBP-EGFP-CLC+mCherry-FRB2), 158 (non-transfected, CLC-FKBP-EGFP+PH-mCherry-FRB2), 125 cells (transfected, CLC-FKBP-EGFP+PH-mCherry-FRB2) from 3 experiments.

(c) Confocal images of HeLa cells expressing FKBP-EGFP-CLC and PH-mCherry-FRB×2 stained with anti-transferrin receptor (TfR) antibody without permeabilization. *n* = 3 experiments. Scale 50 μm.

(d) Fluorescence intensities of surface TfRs staining normalized by non-transfected cells in the same sample were compared between non-transfected and transfected cells. *n* = 131 (non-transfected, FKBP-EGFP-CLC+PH-mCherry-FRB2), 130 (transfected, FKBP-EGFP-CLC+PH-mCherry-FRB2), 153 (non-transfected, FKBP-EGFP-CLC+mCherry-FRB2), 135 (transfected, FKBP-EGFP-CLC+mCherry-FRB2), 121 (non-transfected, CLC-FKBP-EGFP+PH-mCherry-FRB2), 118 cells (transfected, CLC-FKBP-EGFP+PH-mCherry-FRB2) from 3 experiments.

For box plots, box is interquartile range, center line is median, center circle is mean, whiskers are minimum and maximum data points with a coefficient value of 1.5. An unpaired t test was used.

Source data are provided as a Source Data file.

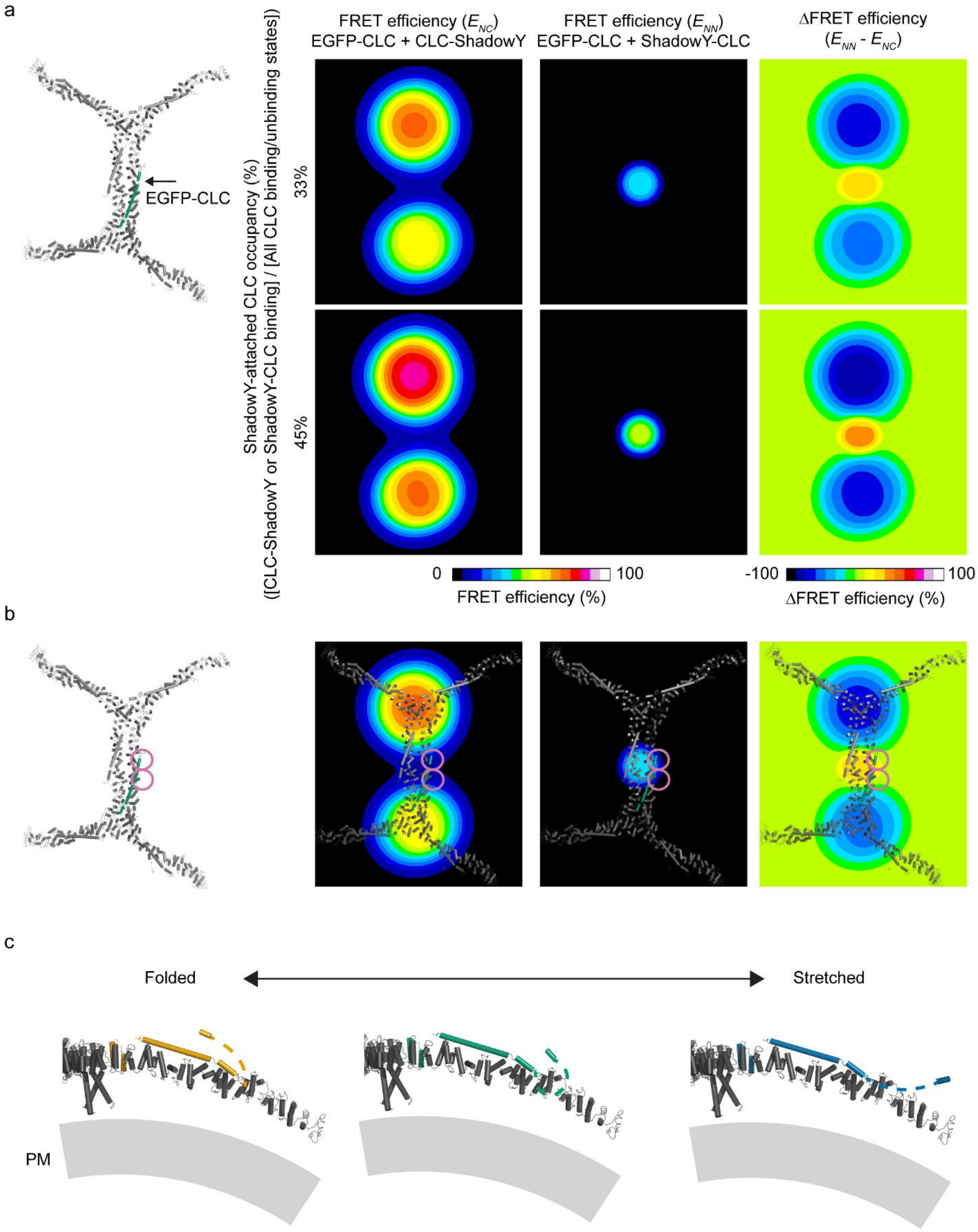

Supplementary Figure 18: FRET simulations at various CLC N-terminal positions in relation to the heavy chain proximal leg domain.

(a) Simulations of FRET efficiency between EGFP-CLC (indicated by arrow in a structural model) and CLC-ShadowY locating at the five closest heavy chain binding positions (left column; E_{NC}), or between EGFP-CLC and ShadowY-CLC locating on the opposite side (center column; E_{NN}). FRET efficiencies were calculated by moving N-terminal position of EGFP-CLC at 33% (top) or 45% (bottom) occupancy rates in ShadowY probes. Right column shows the differences in FRET efficiencies between E_{NN} and E_{NC}. The model is based on PDB 3LVG and 6WCJ. 3LVG is overlaid with 6WCJ.

(b) Structural models are overlaid with simulation results at 33% occupancy rate. Magenta circles indicate the expected N-terminal positions of CLC.

(c) Structural models of possible CLC conformations with various folding states in N-terminal domain. The models are based on PDB 3LVG.

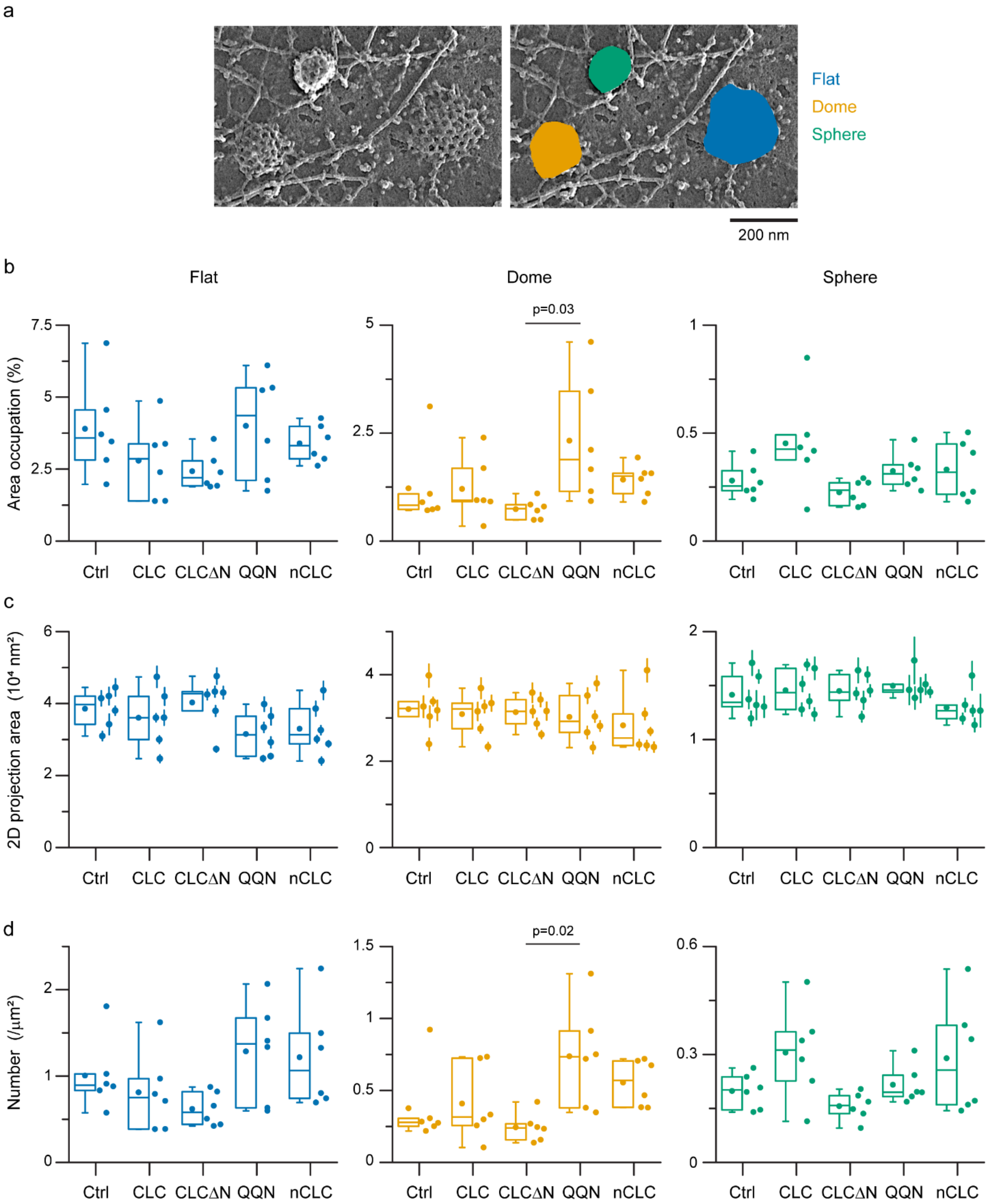

Supplementary Figure 19: PREM analysis on lattice structures.

PREM data from Fig. 2d-f and Supplementary Figs. 5 and 9 were analyzed. Non-transfected cells (control) in the same samples were imaged and analyzed (*n* = 6 cells from 3 experiments). Two-

dimension area of single CCS were manually segmented and measured. The average measured area (mean ± SE) = 156 ± 16 (Ctrl), 206 ± 43 (EGFP-CLC), 312 ± 26 (EGFP-CLCΔN), 143 ± 32 (EGFP-QQN), and 159 ± 27 μm^2 (EGFP-nCLC).

(a) A PREM image of an EGFP-CLC expressing cell. Right image shows segmentation example. Scale 200 nm.

(b-d) Membrane area occupation against the total analyzed membrane area (b), two-dimension projection area (c), and density (d) of flat, domed, and sphere CCSs were compared. One-way ANOVA, then Tukey's test. Each dot is from one cell and errors are SE. For box plots, box is interquartile range, center line is median, center circle is mean, whiskers are minimum and maximum data points with a coefficient value of 1.5. Source data are provided as a Source Data file.
