# Supplementary material for: A conformational switch in clathrin light chain regulates lattice structure and endocytosis at the plasma membrane of mammalian cells
Source: Nat Commun. 2023 Feb 9;14:732. doi: 10.1038/s41467-023-36304-7 (PMC9911608; doi:10.1038/s41467-023-36304-7)
Supplement: Supplementary file 3 — Description of Additional Supplementary Files [file 41467_2023_36304_MOESM3_ESM.pdf]

Description of Additional Supplementary Files

File Name: Supplementary Data 1
Description: Plasmid list used in this study.
