# Supplementary material for: A conformational switch in clathrin light chain regulates lattice structure and endocytosis at the plasma membrane of mammalian cells
Source: Nat Commun. 2023 Feb 9;14:732. doi: 10.1038/s41467-023-36304-7 (PMC9911608; doi:10.1038/s41467-023-36304-7)
Supplement: Supplementary file 5 — Reporting Summary [file 41467_2023_36304_MOESM5_ESM.pdf]

Corresponding author(s): Dr. Justin W. Taraska
Last updated by author(s): Jan 20, 2023

Reporting Summary

Nature Portfolio wishes to improve the reproducibility of the work that we publish. This form provides structure for consistency and transparency in reporting. For further information on Nature Portfolio policies, see our Editorial Policies and the Editorial Policy Checklist.

Statistics

For all statistical analyses, confirm that the following items are present in the figure legend, table legend, main text, or Methods section.

n/a	Confirmed	
☐	☒	The exact sample size (*n*) for each experimental group/condition, given as a discrete number and unit of measurement
☒	☐	A statement on whether measurements were taken from distinct samples or whether the same sample was measured repeatedly
☐	☒	The statistical test(s) used AND whether they are one- or two-sided<br>*Only common tests should be described solely by name; describe more complex techniques in the Methods section.*
☒	☐	A description of all covariates tested
☐	☒	A description of any assumptions or corrections, such as tests of normality and adjustment for multiple comparisons
☐	☒	A full description of the statistical parameters including central tendency (e.g. means) or other basic estimates (e.g. regression coefficient) AND variation (e.g. standard deviation) or associated estimates of uncertainty (e.g. confidence intervals)
☐	☒	For null hypothesis testing, the test statistic (e.g. *F*, *t*, *r*) with confidence intervals, effect sizes, degrees of freedom and *P* value noted<br>*Give P values as exact values whenever suitable.*
☒	☐	For Bayesian analysis, information on the choice of priors and Markov chain Monte Carlo settings
☒	☐	For hierarchical and complex designs, identification of the appropriate level for tests and full reporting of outcomes
☒	☐	Estimates of effect sizes (e.g. Cohen's *d*, Pearson's *r*), indicating how they were calculated

Our web collection on statistics for biologists contains articles on many of the points above.

Software and code

Policy information about availability of computer code

Data collection	Nikon (NIS) Elements AR versions 5.20, Leica LAS X Microscopy Software(version 3.5.6), i3 Slidebook6 were used to collect the fluorescence images. SerialEM (version 3.0.2) was used to acquire EM montages.
Data analysis	IMOD (version 4.9.8) was used to process EM data. MatLab (version R2019b) was used for correlating the images. The codes used in this study are specific to lab file formatting and are available in Figshare at https://doi.org/10.25444/nhlbi.c.5405490.v1. A statement of code availability is given in the methods section. ImageJ 1.53c was used to process and analyze FLIM, TIRF, pol-TIRF, confocal, and EM images, and ImageJ plugin TrackMate was used to analyze live-cell data. Origin 2016 was used for statistical analysis.

For manuscripts utilizing custom algorithms or software that are central to the research but not yet described in published literature, software must be made available to editors and reviewers. We strongly encourage code deposition in a community repository (e.g. GitHub). See the Nature Portfolio guidelines for submitting code & software for further information.

Data

Policy information about availability of data

All manuscripts must include a data availability statement. This statement should provide the following information, where applicable:

- Accession codes, unique identifiers, or web links for publicly available datasets
- A description of any restrictions on data availability
- For clinical datasets or third party data, please ensure that the statement adheres to our policy

The raw data generated in this study has been deposited in Figshare at https://doi.org/10.25444/nhlbi.c.6259170. The remaining data are available in the Article or Supplementary Information files. The processed data is available in Supplementary information/Source data provided with this paper.
Atomic coordinates of previously determined X-ray or Cryo-EM structures are available in the PDB under the following accession codes: 3LVG (clathrin heavy chains and light chains), [https://doi.org/10.2210/pdb3LVG/pdb], 6WCJ (clathrin cage), [https://doi.org/10.2210/pdb6WCJ/pdb], and 4KW4 (GFP), [https://doi.org/10.2210/pdb4KW4/pdb].

Human research participants

Policy information about studies involving human research participants and Sex and Gender in Research.

Reporting on sex and gender	N/A
Population characteristics	N/A
Recruitment	N/A
Ethics oversight	N/A

Note that full information on the approval of the study protocol must also be provided in the manuscript.

Field-specific reporting

Please select the one below that is the best fit for your research. If you are not sure, read the appropriate sections before making your selection.

☒ Life sciences ☐ Behavioural & social sciences ☐ Ecological, evolutionary & environmental sciences

For a reference copy of the document with all sections, see nature.com/documents/nr-reporting-summary-flat.pdf

Life sciences study design

All studies must disclose on these points even when the disclosure is negative.

Sample size	No statistical method was used to predetermine sample size. Sample sizes were kept as large as possible given experimental constraints, and in ranges similar to previous similar studies from the lab. For CLEM, more than six cells from at least three independent experiments were imaged and more than 50 clathrin lattices were analyzed for each cells. For confocal and FLIM imaging, more than 15 cells were imaged from at least three independent experiments. For live-cell TIRF experiments, five cells from at least three independent experiments were imaged from which more than 30 clathrin spots were analyzed for each cell. For weseter blotting, transfection, protein isolation, SDS-PAGA/blotting were repeated three times. Sample sizes are provided in figure legends and the Data source file.
Data exclusions	For FRET-CLEM, cells with larger than 2.1 ns in cell-average fluorescence lifetime were excluded due to not confirming the expression of ShadowY probes. For live cells TIRF, fluorescnce spots which were not satidfied the criteria described in Methods were excluded.
Replication	Repetitive biologically independent experiments were done to confirm consistency of results. All attempts at replication were successful for those experiments. Sample sizes (number of cells, structures or spots analyzed) and number of biologically independent experiments with consistent results are indicated in the corresponding figure legends.
Randomization	Not done in this study. In each experiments, there were defined groups e.g. donor only vs. donor and acceptor, wild type vs. mutants, or control vs. specific drug, and randomization was not necessary.
Blinding	Data collection were not performed blind to the conditions of the experiments. Data collection for fluorescent, electron, and correlative microscopy imaging purposes involved selection of random fields of view, the parameters we measured are not clearly distinguished by the human eye. This implies that the experimenters were inherently blinded to the outcome of each experiment, and no other procedures were necessary. Blinding during data analysis was not possible because all data was analyzed by the first author.

Reporting for specific materials, systems and methods

We require information from authors about some types of materials, experimental systems and methods used in many studies. Here, indicate whether each material, system or method listed is relevant to your study. If you are not sure if a list item applies to your research, read the appropriate section before selecting a response.

Materials & experimental systems

n/a	Involved in the study	
☐	☒	Antibodies
☐	☒	Eukaryotic cell lines
☒	☐	Palaeontology and archaeology
☒	☐	Animals and other organisms
☒	☐	Clinical data
☒	☐	Dual use research of concern

Methods

n/a	Involved in the study	
☒	☐	ChIP-seq
☒	☐	Flow cytometry
☒	☐	MRI-based neuroimaging

Antibodies

Antibodies used

For western blot
1:5000, polyclonal rabbit anti-clathrin light chain a and b (Millipore, AB9884)
https://www.emdmillipore.com/US/en/product/Anti-Clathrin-Light-Chain-Antibody,MM_NF-AB9884?ReferrerURL=https%3A%2F%2Fwww.google.com%2F
1:1000, polyclonal rabbit anti-clathrin light chain a (Sigma, HPA050918)
https://www.sigmaaldrich.com/US/en/product/sigma/hpa050918
1:500, monoclonal mouse anti-clathrin light chain b (Abnova, H00001212-M01, clone 4B12-1E3)
http://www.abnova.com/products/products_detail.asp?catalog_id=H00001212-M01
1:2000, polyclonal rabbit anti-beta actin-HRP (Cell Signaling, 5215)
https://www.cellsignal.com/products/antibody-conjugates/b-actin-13e5-rabbit-mab-hrp-conjugate/5125
1:2000, polyclonal goat anti-mouse IgG-HRP (Jackson ImmunoResearch Labs, 115-035-174)
https://www.jacksonimmuno.com/catalog/products/115-035-174
1:2000, monoclonal mouse anti-rabbit IgG-HRP (Jackson ImmunoResearch Labs, 211-032-171)
https://www.jacksonimmuno.com/catalog/products/211-032-171

For immunocytochemistry
1:50 monoclonal mouse anti-transferrin receptor (Santa Cruz, sc-65877, clone b3/25)
https://www.scbt.com/p/cd71-antibody-b3-25
1:500 polyclonal goat anti-mouse IgG-Alexa Fluor 647 (Invitrogen, A-21237)
https://www.thermofisher.com/antibody/product/Goat-anti-Mouse-IgG-H-L-Cross-Adsorbed-Secondary-Antibody-Polyclonal/A-21237

Validation

Rabbit anti-clathrin light chain a and b (Millipore, AB9884): the manufacturer's website mentions that the antibody has been tested for WB and has species reactivity for human and rat. References: PMID: 23761069 and 28231468.
Rabbit anti-clathrin light chain a (Sigma, HPA050918): the manufacturer's website mentions that the antibody has been tested for IHC and WB and has species reactivity for human. References: PMID: 28171750.
Mouse anti-clathrin light chain b (Abnova, H00001212-M01, clone 4B12-1E3): the manufacturer's website mentions that the antibody has been tested for ELISA, IF, IHC, and WB and has species reactivity for human. References: PMID: 28171750.
Mouse anti-transferrin receptor (Santa Cruz, sc-65877, clone b3/25): the manufacturer's website mentions that the antibody has been tested for IF, IP, and WB and has species reactivity for human. References: PMID: 21471245, 28004827, 28067430, 29437695, and 29570778.

Eukaryotic cell lines

Policy information about cell lines and Sex and Gender in Research

Cell line source(s)	HeLa: ATCC #CCL-2; RRID: CVCL_0030<br>SK-MEL-2: ATCC #HTB-68; RRID: CVCL_0069
Authentication	No
Mycoplasma contamination	All cell lines were routinely tested for the mycoplasma contamination and were negative.
Commonly misidentified lines (See ICLAC register)	No commonly misidentified lines were used in this study.
